# Indoximod opposes the immunosuppressive effects mediated by IDO and TDO via modulation of AhR function and activation of mTORC1

**DOI:** 10.18632/oncotarget.27646

**Published:** 2020-06-23

**Authors:** Erik L. Brincks, James Adams, Lifu Wang, Benjamin Turner, Agnieszka Marcinowicz, Jiyuan Ke, Michael Essmann, Lucía M. Mautino, Clarissa Van Allen, Sanjeev Kumar, Nicholas Vahanian, Charles Link, Mario R. Mautino

**Affiliations:** ^1^NewLink Genetics Corporation, Ames, IA, USA; ^2^Lumos Pharma, Inc., Ames, IA, USA

**Keywords:** indoximod, IDO, TDO, AhR, mTORC1

## Abstract

Indoximod has shaped our understanding of the biology of IDO1 in the control of immune responses, though its mechanism of action has been poorly understood. Previous studies demonstrated that indoximod creates a tryptophan (Trp) sufficiency signal that reactivates mTOR in the context of low Trp concentrations, thus opposing the effects caused by IDO1. Here we extend the understanding of indoximod’s mechanism of action by showing that it has pleiotropic effects on immune regulation. Indoximod can have a direct effect on T cells, increasing their proliferation as a result of mTOR reactivation. Further, indoximod modulates the differentiation of CD4^+^ T cells via the aryl hydrocarbon receptor (AhR), which controls transcription of several genes in response to different ligands including kynurenine (Kyn). Indoximod increases the transcription of *RORC* while inhibiting transcription of *FOXP3*, thus favoring differentiation to IL-17-producing helper T cells and inhibiting the differentiation of regulatory T cells. These indoximod-driven effects on CD8^+^ and CD4^+^ T cells were independent from the activity of IDO/TDO and from the presence of exogenous Kyn, though they do oppose the effects of Kyn produced by these Trp catabolizing enzymes. Indoximod can also downregulate expression of IDO protein *in vivo* in murine lymph node dendritic cells and *in vitro* in human monocyte-derived dendritic cells via a mechanism that involves signaling through the AhR. Together, these data improve the understanding of how indoximod influences the effects of IDO, beyond and distinct from direct enzymatic inhibition of the enzyme.

## INTRODUCTION

Indoleamine 2,3-dioxygenase (IDO1) plays an important role in the regulation of acquired local and peripheral immune tolerance in normal and pathological scenarios [1]. IDO-expressing cells are found constitutively in many non-transformed tissues, including epididymis, gut (distal ileum and colon), lymph nodes, spleen, thymus, and lungs, where it seems to regulate local inflammation and moderate response to foreign or uncommon non-pathological antigens. IDO activity is also found constitutively at the maternal–fetal interface, where it plays a critical role in inducing maternal immune tolerance to the father-derived allogeneic antigens expressed by the fetus [2–5]. In cancer, IDO1 can be expressed either directly by the tumor cells or induced indirectly in host antigen presenting cells by the tumor. IDO1 expression by tumor cells has been associated with significantly worse clinical prognosis and reduced survival in malignant melanoma [6, 7], pancreatic cancer [8], ovarian cancer [9], both pediatric [10] and adult acute myelogenous leukemia [11, 12], colorectal cancer [13], prostate cancer [14], endometrial cancer [15], and others [16, 17].

The immunosuppressive effects of IDO1 are mediated by the enzymatic conversion of L-tryptophan (Trp) into N-formyl-kynurenine, which is then metabolized to L-kynurenine (Kyn) and subsequently to many other metabolites along the Kyn pathway [18]. Two functionally-related proteins that catalyze the same biochemical reaction are IDO2 [19–21] and tryptophan 2,3-dioxygenase (TDO) [22]. TDO is expressed constitutively by the liver, but also in in certain tumors, which suggests a possible functional role in cancer immunosuppression which can be independent or complementary to that of IDO1 [16, 23].

The cellular pharmacodynamic effects of IDO1 activity include the inhibition of antigen-specific CD8^+^ T cell proliferation [24–26], stimulation of differentiation of naïve CD4^+^ T cells to FoxP3^+^ regulatory T cells (Tregs) [27, 28], the activation of Tregs [29], and the recruitment of MDSC to the tumor [30]. Both degradation of Trp and generation of Kyn (and its downstream metabolites) elicit independent biochemical signals that mediate these cellular effects and modulate acquired immunologic tolerance during inflammatory processes.

Low levels of Trp are sensed by the stress kinase GCN2, which contains a regulatory domain that binds aminoacyl-free tRNA [31, 32]. When GCN2 binds uncharged tRNA, its kinase domain is activated and phosphorylates the ribosomal initiation factor eukaryotic initiation factor 2 alpha (eIF2α) [33], preventing the translation of most mRNA species, except for some genes involved in coordination of a cellular response to protect and recover from the nutrient deficiency [32–34]. Activation of GCN2 also results in inactivation of MAP4K3/GLK, a protein that integrates the amino acid sufficiency and deficiency sensing signals [35, 36]. Trp deficiency created by IDO also inhibits MAP4K3 and its downstream substrate PKCθ, and in the inactivation of mTORC1 [37]. Activation of GCN2 and inactivation of mTORC1 are responsible for mediating the anergy and arrest of CD8^+^ T cell proliferation as well as contributing to the differentiation, activation, and maintenance of the suppressive state of Tregs [28, 31].

In addition to depleting Trp, IDO1 and TDO initiate the generation of a series of downstream metabolites along the Kyn pathway (Kyns). Kyns are immunologically-active ligands for the aryl hydrocarbon receptor (AhR) [38, 39]. The AhR is a ligand-activated transcription factor, which upon binding to its ligands translocates to the nucleus. There it binds to the its partner aryl hydrocarbon nuclear receptor translocator (ARNT) and regulates transcription of a number of genes containing DRE/XRE control sequences, in concert with other transcription factors [40]. Examples of functionally-relevant genes that can be regulated by AhR include *FOXP3* [38, 40, 41], a key transcription factor controlling the function of Tregs; *RORC*, a transcription factor associated with Th17 cell programming [42]; and *IDO1* [43, 44]. The transcriptional function of AhR on a specific promoter is highly dependent on the small-molecule ligand, interactions with other transcription factors, and the metabolic status of the cell. For example, it has been reported that Kyn or 2,3,7,8- tetrachlorodibenzo-p-dioxin (TCDD) can drive differentiation of naïve CD4^+^ T cells into FoxP3^+^ Tregs in an AhR-dependent manner [38]. Other AhR ligands such as FICZ can skew CD4^+^ T cell differentiation towards an IL-17-producing helper T cell (TH17) [38, 41, 45]. The role of AhR in CD8^+^ effector T cells is less well understood, and its influence on effector T cell function and T cell memory are unclear. Moreover, activation of AhR also results in promoting a tolerogenic phenotype on dendritic cells (DC) and stimulation with TCDD or Kyn was shown to induce IDO expression in DCs [39, 43, 46], suggesting a feed-forward loop of immunosuppressive Trp metabolism.

Based on the mechanisms described above, inhibition of IDO enzymatic activity during cancer therapy is desirable to restore immune reactivity against tumors and to prevent the re-establishment of immune suppression following the active immunization processes that are triggered by tumor vaccination, chemotherapy, radiotherapy, or checkpoint inhibitor therapy.

One of the first IDO pathway inhibitors studied in preclinical models has been 1-methyl-DL-tryptophan (1mT), a racemic mixture of enantiomers, which was shown to mediate immune-dependent rejection of allogeneic fetuses in mice [4] and immune-dependent enhancement of antitumor activity of chemotherapy and radiotherapy [47, 48]. Both isomers are capable of restoring T-cell proliferation in an MLR assay with IDO^+^ dendritic cells as the stimulator cells, or in syngeneic antigen-dependent T-cell proliferation assays using IDO^+^ dendritic cells isolated from tumor-draining lymph nodes (TDLN) [49]. Interestingly, both isolated isomers show different potency in this assay, with indoximod being more potent (half maximal effective concentration [EC_50_] ~40 μM) than L1mT (EC_50_ = 80 μM–100 μM) or the racemic mixture (80 μM–100 μM) [49]. L1mT is a competitive inhibitor and substrate of IDO1 enzymatic activity (Ki = 19 μM) in cell-free assays using purified recombinant IDO1 enzyme [49], and in tumor cells treated with INFγ or in tumor cell lines transfected with expression vectors that encode IDO1 under the control of an heterologous promoter [49]. Puzzingly, indoximod does not inhibit IDO1 enzymatic activity *in vitro*, but somehow it mimics the biological consequences of IDO1 inhibition *in vivo* or in cell-based assays. This suggests that IDO1 may not be the primary molecular target of indoximod; but rather, that indoximod exerts its pharmacological effect by countering the downstream effects of IDO activity.

It was previously shown that indoximod does not inhibit the effects of IDO1 by inhibiting the activation of GCN2 triggered by Trp deficiency [37]. Instead, under conditions of Trp deficiency, indoximod creates an artificial Trp-sufficiency signal which is capable of reactivating MAP4K3, as evidenced by phosphorylation of its substrate PKCθ, and subsequently reactivating mTORC1 as evidenced by the increase in pS6K phosphorylation [37]. Therefore, it is currently hypothesized that indoximod acts by creating an artificial Trp-sufficiency signal that reactivates the function of mTORC1 under conditions of Trp-deficiency [50]. The implication of this mechanism is that indoximod should also be able to reactivate mTOR under immunosuppressive conditions imposed by either IDO or TDO expression, thereby making indoximod a dual IDO/TDO inhibitor.

In addition, indoximod can mediate the AhR-dependent induction of *CYP1A1* and of reporter genes driven by an AhR-dependent promoter [51]. This suggests that indoximod could potentially be an antagonist of the Kyn/AhR interaction (i.e., a competitive inhibitor of Kyn), thus blocking the downstream immunosuppressive effects of Kyn on T cells. Intriguingly, the stereoisomer 1-methyl-L-tryptophan was unable to mediate induction of AhR-regulated genes, providing an a potential explanation for the superior activity of indoximod over its stereoisomer in T cell reactivation assays [51].

In this work, we have expanded the current understanding of the mechanism of action of indoximod. Our data suggest that indoximod restores proliferation of T cells in conditions of nutrient depletion created via IDO or TDO by reactivating the mTOR pathway in T cells. We also demonstrate that indoximod-driven modulation of AhR signaling influences the differentiation of CD4^+^ T cells away from a regulatory (Treg) phenotype and toward helper T cell phenotype. Additionally, we show that indoximod can influence the expression of IDO1 in dendritic cells, which appears to be maintained via a AhR-dependent mechanism [43].

## RESULTS

### Indoximod stimulates CD8^+^ T cell proliferation in an IDO/TDO-independent manner

The original characterizations of indoximod as an inhibitor of the IDO pathway demonstrated that indoximod could counteract the effects of IDO function by stimulation of CD8^+^ T cell proliferation in the IDO^+^ TDLN environment [31], in co-culture assays with IDO^+^ plasmacytoid dendritic cells (pDCs) isolated from TDLNs [52], or in co-cultures with IDO^+^ allogeneic human moDCs [26]. However, since indoximod does not inhibit the enzymatic activity of purified IDO1 *in vitro* or in cell-based assays [49], we hypothesized that indoximod could restore T cell proliferation by opposing the effects of IDO-mediated deprivation of Trp instead of mediating direct or indirect inhibition of IDO enzymatic or non-enzymatic activity in the IDO^+^ pDCs. To test this hypothesis, we co-cultured TDO-expressing SW48 human colorectal adenocarcinoma cells [53] with human CD8^+^ T cells stimulated to proliferate by anti-CD3/CD28 beads. By utilizing a TDO-driven depletion of Trp, and by eliminating dendritic cells as stimulators of T cell proliferation, this system eliminated the contribution of IDO to the suppression of T cell proliferation and allowed us to investigate whether indoximod’s mechanism of action is taking place directly on the T cell.

**Figure 1:**
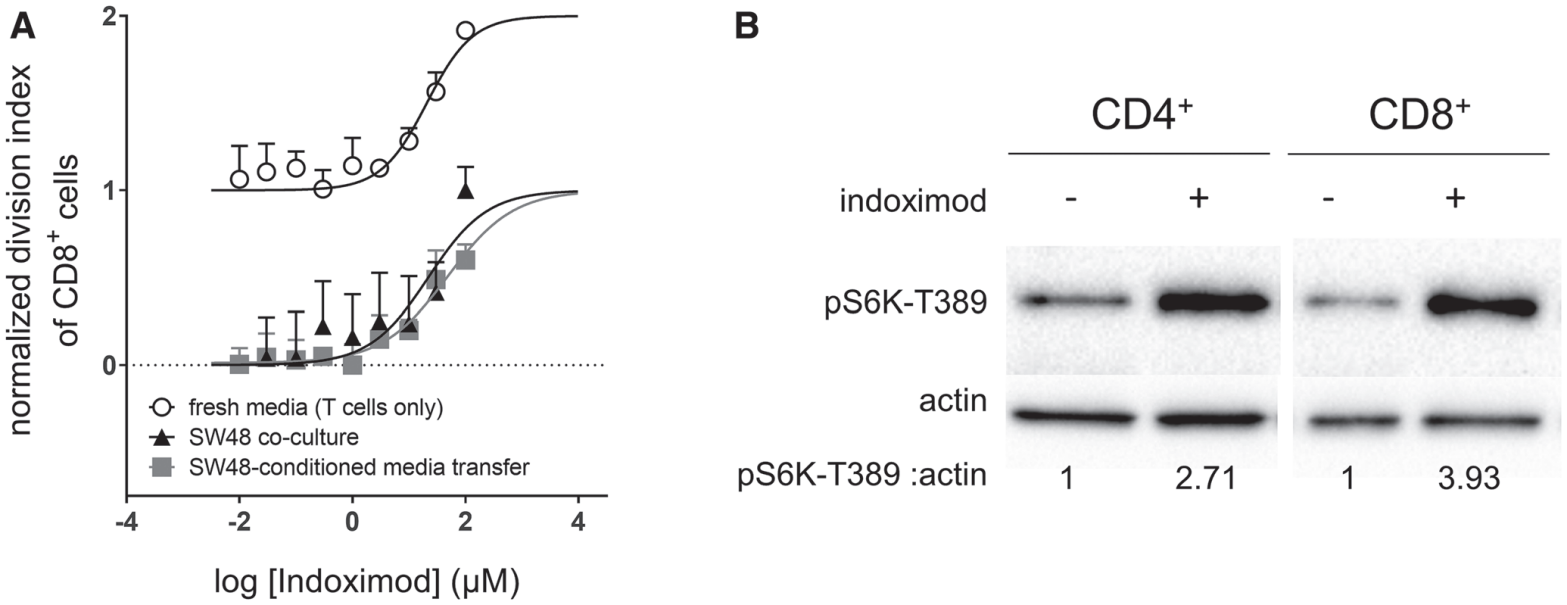
Indoximod augments CD8^+^ T cell proliferation in both nutrient depleted and nutrient replete conditions. (**A**) human CD8^+^ T cells were activated with anti-CD3/CD28 beads and allowed to proliferate in varying culture conditions in the presence of increasing concentrations of indoximod. T cells were co-cultured with TDO-expressing SW48 cells (black triangles), or in media conditioned by TDO-expressing SW48 cells (gray squares), or in unconditioned/fresh media (open circles). Proliferation of T cells was measured by the dilution of cytoplasmic dye via flow cytometry. The division index was calculated for each sample, and normalized to minimum (0, no stimulation beads) and maximum (1, proliferation in fresh media with no indoximod) values. Plots are representative data of 3 or more experimental runs for each condition. No statistical difference in the EC_50_ values was observed when comparing the culture conditions (*p* > 0.05). (**B**) primary human PBMC were enriched for CD4^+^ or CD8^+^ T cells by negative magnetic selection. These enriched populations were cultured overnight in Trp-free RPMI media. Cells were stimulated with indoximod for 5 h and mTOR activation was assessed by pS6K expression in western blot. Ratio of pS6K: actin determined by densitometry is shown below each lane.

In these cultures, the TDO expressed by the SW48 cells converted tryptophan into kynurenine, thereby depleting the local concentrations of tryptophan necessary for optimal T cell proliferation. In the absence of indoximod and under conditions of no inhibition of the TDO pathway T-cell proliferation is blocked (Figure 1A). Addition of indoximod restores CD8^+^ T cell proliferation in co-cultures with an EC_50_ of 23.2 μM (95% CI: 14.6 μM to 36.7 μM) to levels observed in control cultures with T cells in fresh media and no indoximod (Figure 1A, black triangles). Of note, this indoximod effect on T cells was apparently independent of indoximod-driven modulation of TDO activity, as the depletion of tryptophan and production of kynurenine was consistent regardless the concentration of indoximod added (Supplementary Figure 1). To verify a TDO-independent effect of indoximod, T cell proliferation was measured at different concentrations of indoximod, in cultures containing media conditioned by SW48 cells for 48 hours (i.e., partially depleted from tryptophan through conversion to kynurenine). Similar to the direct co-culture system, indoximod restored CD8^+^ T cell proliferation to levels similar to what was observed in control cultures of fresh media, with an EC_50_ of 41.4 μM (95% CI: 31.1 μM to 55.4 μM; Figure 1A, gray squares). This observation indicates that indoximod has a direct stimulatory effect on the proliferation of T cells and supports the hypothesis that indoximod opposes the downstream effects of both IDO and TDO. Furthermore, it suggests that a molecular target of indoximod resides in the T cell rather than in the IDO- or TDO-expressing cell.

To further investigate whether indoximod has T cell stimulatory effects that are independent of the conditions imposed by IDO and TDO function, we cultured stimulated CD8^+^ T cells in fresh media in the presence of different concentrations of indoximod. Under these conditions, indoximod displayed no obvious proliferation-limiting toxicity. In fact, T cell proliferation was enhanced above the rate observed in cultures containing fresh media without indoximod with an estimated EC_50_ of 25.7 μM (95% CI: 13.5 μM to 36.9 μM; Figure 1A, open circles).

To test whether this IDO/TDO-independent T cell stimulatory effect of indoximod was mediated by activation of mTOR, we tested the ability of indoximod to activate mTOR in primary human T cells cultured overnight in Trp-deficient media. When T cells were treated with indoximod, both CD4^+^ and CD8^+^ T cells had activated mTOR, as evidenced by the expression of pS6K (Figure 1B). This mTOR activation in the T cells was similar to the indoximod-driven mTOR activation previously observed in MCF7 cells cultured under Trp-deficient conditions [37].

Taken together, these results suggest that indoximod can stimulate mTOR on the T cells, thus opposing the inhibition of mTOR created by Trp deprivation that takes place in microenvironments created by IDO or TDO-expressing cells. Of note, stimulation of T cell proliferation in fresh media suggests that activation of mTOR by indoximod could take place even under normal nutrient conditions.

### Indoximod contributes to the *in vivo* modulation of IDO expression and activity

**Figure 2:**
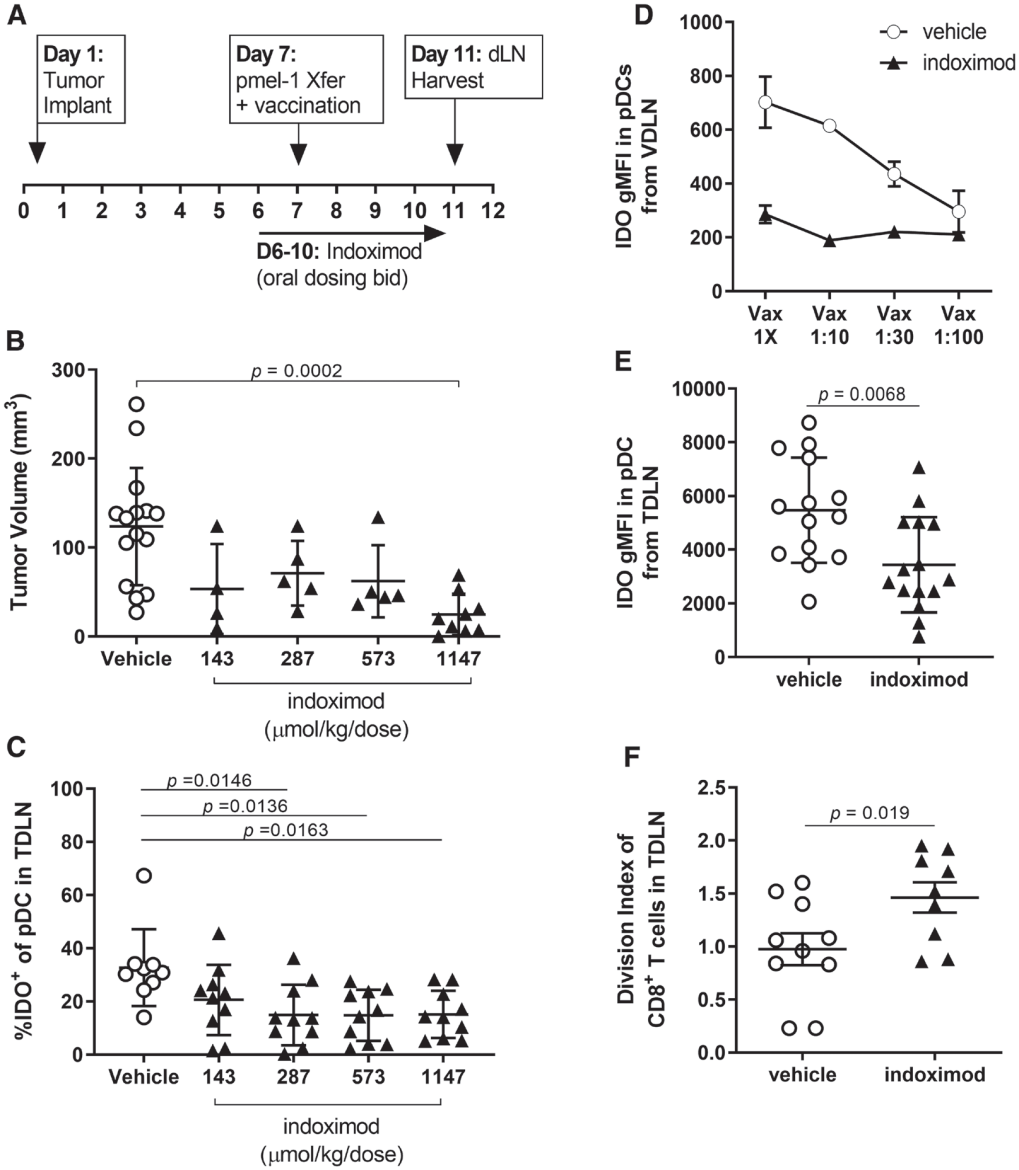
Indoximod downregulates IDO protein expression in pDCs from TDLN and VDLN (**A**) Schematic overview of experimental design. Immunotherapy of established B16F10 melanoma consisted of the adoptive transfer of pmel-1 T cells and gp100/CpG vaccination plus escalating doses of indoximod administered BID by oral gavage. (**B**) Antitumor response as function of indoximod dose, on day 11 after tumor implant. Statistical significance was determined using ANOVA (overall significance *p* = 0.0003) with follow up Dunnett’s multiple comparisons test comparing each dosing group against the vehicle plus vaccine (VAX) control group (^*^ *p* = 0.0002). (**C**) frequency of IDO^+^ pDC among total pDC in the tumor-draining lymph node of mice treated with the various doses of indoximod. Statistical significance was determined using ANOVA (overall significance *p* = 0.0068) with follow up Tukey’s multiple comparisons test comparing each dosing group against the vehicle control group. (**D**) Tumor-bearing mice were treated with adoptive transfer of tumor-specific T cells plus vaccination on day 7 post-tumor-implantation. Strength of vaccination (dilution factor of vaccine) is plotted on the x-axis. Vaccine-draining lymph nodes (popliteal LN) were harvested on day 11 post-tumor-implantation. Data represent average gMFI of IDO staining in the pDC population (defined as the CD317^+^CD11c^+^ cells) in the VDLN on Day 11 after tumor implantation. Points represent mean values (+/− standard deviation) of *n* = 3 mice in each vaccination group. (**E**) Effect of indoximod treatment on the expression of IDO by pDCs. Data represent the gMFI of IDO in the pDC population defined as the CD317^+^CD11c^+^ cells harvested from the TDLN of each animal on day 11. (**F**) Effect of indoximod treatment of mice on proliferation of dye-labeled pmel-1 CD8^+^ T cells harvested from TDLN. Data represent the average number of divisions by a tumor-specific T cell (identified as CD8^+^CD90.1^+^).

Historical data suggest that indoximod blocks production of kynurenine by IDO expressed in dendritic cells [29, 49], yet paradoxically, studies have demonstrated that indoximod is not an inhibitor of IDO enzymatic activity [49, 54, 55]. Together, these observations suggest that an alternative mechanism is responsible for indoximod’s influence on IDO function in dendritic cells. Regulation of IDO protein expression and function can also be controlled through transcriptional regulation; for example, treatment with indoximod analogs (L-Trp, 1-methyl-L-Trp, MTH-Trp) can modulate IDO expression in a murine tumor cell line [56]. To determine the extent to which indoximod modulates IDO expression in dendritic cells, we examined IDO expression by dendritic cells from the tumor draining lymph nodes of mice bearing established B16F10 tumors treated with an immunotherapy consisting of the adoptive transfer of tumor-specific pmel-1 T cells and gp100 peptide vaccination (per the experimental design outlined in Figure 2A). Consistent with reports using other experimental models [30, 49, 57], the data presented in Figure 2B demonstrate that indoximod dosing improves the anti-tumor effect of this cell-based immunotherapy. The frequency of IDO^+^ pDCs in the TDLN of mice was decreased in dose-dependent fashion that correlated with the observed decreases in tumor size (Figure 2C), with doses of indoximod higher than 287 μmol/kg/dose bid resulting in a significant decrease in the proportion of IDO^+^ pDC in the TDLN.

A follow up experiment sought to determine the extent to which IDO expression is driven by inflammation associated with the immunotherapy regimen, in which the same number of pmel-1 cells were transferred into tumor-bearing mice with a dilution of the gp100/CpG vaccination dose (Figure 2D). Inflammation driven by the vaccine appears to induce IDO1 protein expression in the vaccine-draining lymph node (VDLN), as evidenced by the dose-dependent increase in the intensity of staining for IDO (gMFI).

Of note, indoximod treatment (250 mg/kg/dose bid) of mice receiving this immunotherapy reduced the observed IDO protein expression to levels similar to those observed in unvaccinated mice, regardless of the vaccination dose. Indoximod treatment also resulted in an overall decrease in the amount of IDO protein present in pDCs from the TDLN compared with expression by DCs in mice treated with vehicle control (Figure 2E). This decrease in IDO protein expression correlated with a decrease in immunosuppressive function, as evidenced by the restoration of proliferation by tumor-specific T cells in the TDLN of indoximod-treated mice (Figure 2F).

### Indoximod drives modulation of IDO expression in moDC via AhR

The existence of an autocrine IDO-kynurenine/AhR-IDO positive feedback loop in IDO^+^ dendritic cells that contributes to the maintenance of IDO1 expression has been reported recently. In this mechanism Kyn acts as a ligand of AhR and drives transcription of IDO1 mRNA [43], a feedback loop that can be interrupted by L1mT. Therefore, we investigated whether the mechanism of the indoximod-driven downregulation of IDO protein could involve the AhR/Kyn/IDO axis. To test this hypothesis, we utilized an *in vitro* culture system in which human monocytes were differentiated in culture conditions that typically facilitate their differentiation into IDO^+^ dendritic cells (moDC) [26, 54, 58] in the presence or absence of indoximod (schematic overview in Supplementary Figure 2A).

**Figure 3:**
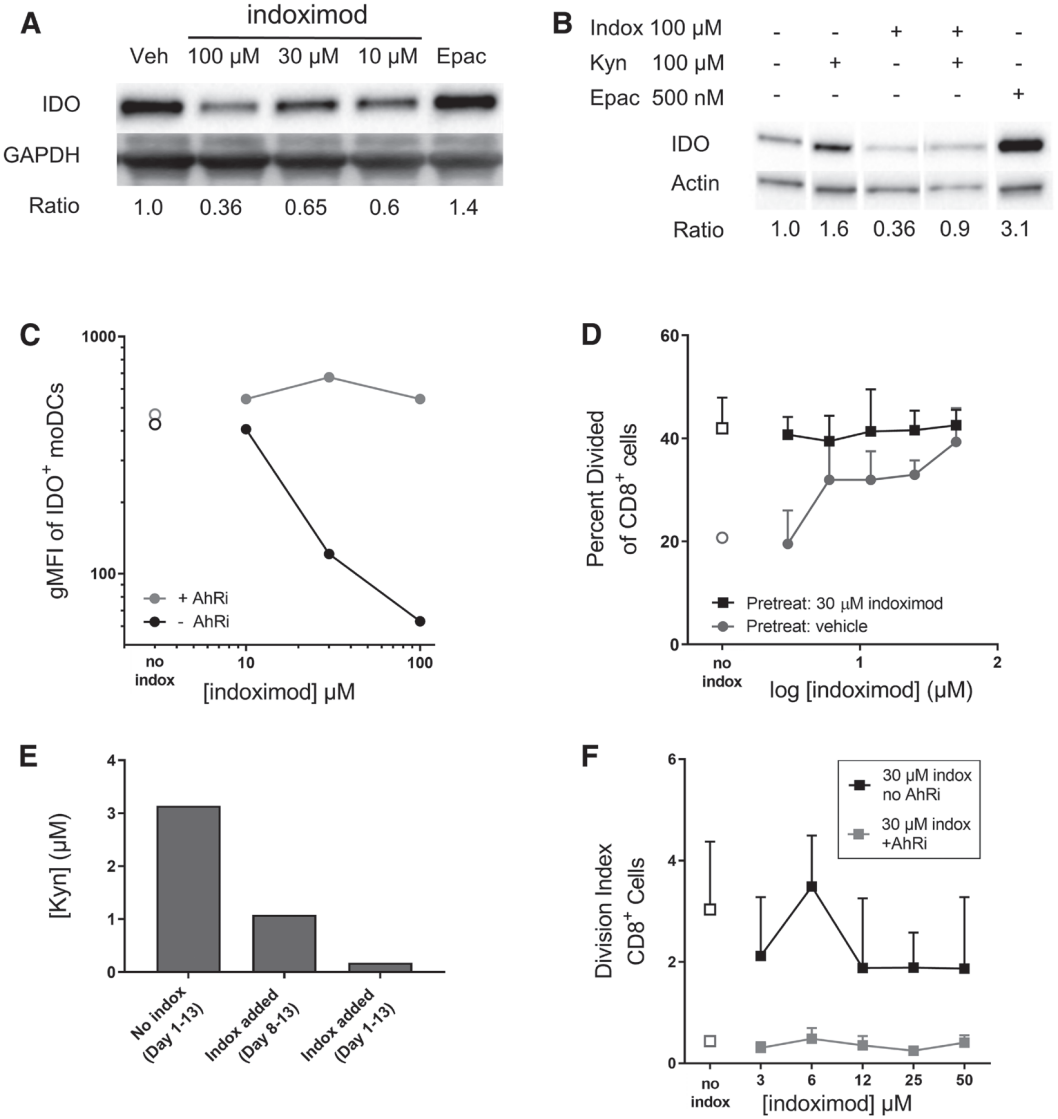
Indoximod downregulates IDO expression in moDC in an AhR-dependent manner. Human monocytes were differentiated into CD11c^+^CD123^+^CD83^+^ dendritic cells (moDC) with or without the addition of indoximod to the differentiation culture on days 1 through 8. Stimulation cocktail of IFNγ, TNFα, IL-1β, IL-6, anti-CD40L, and PGE2 was added during the last 48 h. (**A**) Western blot of cell lysates from differentiated moDC to assess downregulation of IDO protein by various concentrations of indoximod or epacadostat (500 nM). (**B**) Differentiation of moDCs was carried out in the presence of indoximod, Kyn, or both, or epacadostat and IDO protein expression at the end of the differentiation period was assessed by western blot. (**C**) moDC were differentiated in the presence of vehicle (open symbols), indoximod (closed black symbols) or indoximod with the AHR inhibitor GNF351 (500 nM) (grey circles). Values represent the gMFI of IDO^+^ moDCs after subtraction of the isotype control gMFI. (**D**) IDO function in moDC differentiated in the absence (circles) or presence of indoximod (30 μM) (squares) during the moDC differentiation phase (days 1–8), were co-cultured with allogeneic CFSE-labeled CD8^+^ T cells, in the absence (open symbols) or presence of indoximod (1–50 μM) (closed symbols) during the T cell proliferation phase (Days 8–13). (**E**) the functional activity of IDO was assessed by measurement of Kyn levels in the moDC: T cell MLR culture supernatants on Day 13. Bar 1: no indoximod added during moDCs differentiation, maturation or MLR; Bar 2: indoximod absent during moDC differentiation or maturation, but present during the MLR culture at 100 μM; Bar 3: 100 μM indoximod present during moDCs differentiation, maturation and MLR. (**F**) Same MLR as in D, using moDCs differentiated in the presence of 30 μM indoximod and in the presence (gray squares) or absence (black squares) of the 500 nM GNF351 during differentiation of moDC.

The addition of indoximod to the differentiation cultures decreased IDO protein expression in a dose-dependent manner (up to ~65% decrease was observed at 100 μM) as assessed by western blot (Figure 3A). Conversely, the addition of 100 μM Kyn during differentiation increased IDO protein expression, an effect that was counteracted by an equal concentration of indoximod. This suggests that Kyn and indoximod regulate IDO protein expression via a common pathway, and is consistent with the Kyn/AhR-mediated effect on IDO mRNA transcription described by Li *et al*. [43] (Figure 3B). Surprisingly, incubation of moDCs with the potent IDO1 enzymatic inhibitor epacadostat, led to an increase in IDO1 protein level (Figure 3A and 3B).

In complementary analyses, IDO expression was assessed in moDCs by flow cytometry. Consistent with the western blot data, indoximod decreases the average per-cell expression of IDO protein in CD11c^+^CD123^+^CD83^+^ moDC in a dose-dependent fashion, with an estimated EC_50_ of 20 μM (Figure 3C).

To investigate the extent to which AhR is involved in indoximod-mediated downregulation of IDO expression in moDCs, the AhR inhibitor GNF-351 was included in the differentiation cultures, and IDO expression assessed by FACS. The data presented in Figure 3C shows that GNF351 blocked the indoximod-driven reduction of IDO protein, suggesting that indoximod’s influence on the expression of IDO is driven, at least in part, by an AhR-dependent pathway.

### Indoximod drives modulation of IDO function in moDC via AhR

The presence of IDO^+^ APCs in MLR cultures limits the proliferative response of T cells. To assess the extent to which indoximod-driven reductions in IDO protein expression could modulate IDO-driven immunosuppression, moDC differentiated in the absence or presence of 30 μM indoximod were used to stimulate allogeneic T cell proliferation in MLR cultures in absence or presence of different concentration of indoximod (see experimental schematic in Supplementary Figure 2B). Consistent with the indoximod-driven reduction in IDO protein expression in moDC, indoximod-treated moDC promoted higher CD8^+^ T cell proliferation compared to the vehicle-treated control moDC (“no indox” vehicle controls, Figure 3D). To test the ability of indoximod to stimulate T cell proliferation in the presence of active IDO protein, IDO^+^ moDC that had been differentiated in the absence of indoximod were used to stimulate T cell proliferation in MLR cultures with indoximod added during the proliferation stage of the MLR culture. As described before by others [26, 49], addition of indoximod during the proliferation stage of the assay restored CD8^+^ T cell proliferation in a dose-dependent fashion (Figure 3D, gray circles). However, when indoximod had been added during the differentiation of the moDC, T cells proliferated at their maximum division rate, even in the absence of indoximod; further, the subsequent addition of indoximod during the T cell proliferation cultures provided no additional proliferation benefit to the T cells (Figure 3D, black squares). The indoximod-driven modulation of IDO protein and T cell proliferation was mirrored in the assessments of kynurenine from the MLR cultures, where the addition of indoximod resulted in decreased kynurenine production (Figure 3E). Of note, addition of indoximod during moDC differentiation resulted in a marked reduction of Kyn in the supernatant, compared to when indoximod was absent during differentiation of moDCs but present during the MLR cultures. The indoximod-driven reduction of Kyn was observed consistently in four human donors for the MLR cultures, indicating that this was not a donor-specific effect (Supplementary Figure 3).

To further assess the involvement of AhR in indoximod’s effect on IDO protein function and stimulation of T cell proliferation, moDC differentiation and subsequent MLR cultures were carried out with the addition of 30 μM indoximod and in the presence or absence of the AhR inhibitor GNF351 (500 nM). Blocking AhR activity during moDC differentiation resulted in greatly reduced T cell proliferation (Figure 3F), an observation that is consistent with the increased IDO levels observed in the presence of GNF351 during moDCs differentiation (Figure 3C). Importantly, the inhibition of T-cell proliferation by AhR inhibitors was not the result of direct toxicity of the AhR inhibitor as the inhibitor was not present during the T cell proliferation phase of the assay. Additionally, T cells stimulated exogenously (by beads in the absence of moDCs) proliferated normally in the presence of AhR inhibitor, indoximod, or both (Supplementary Figure 4). Taken together, these data are consistent with a mechanism in which indoximod could inhibit the activity of AhR-dependent transcription of IDO1 (perhaps in combination with other transcription factors), and oppose the positive feedback loop mediated by Kyn/AhR on IDO1 expression. In this model, indoximod activity would result in reduced expression of IDO1 protein, reduced production of Kyn, and altered phenotype of moDCs from immunosuppressive to immunostimulatory.

### Indoximod modulates AhR-mediated transcription of target genes

Extending the observation that an AhR inhibitor could block the indoximod-driven modulation of IDO protein expression, we next explored if indoximod is an AhR ligand capable of driving the transcription of genes controlled by the AhR activity.

**Figure 4:**
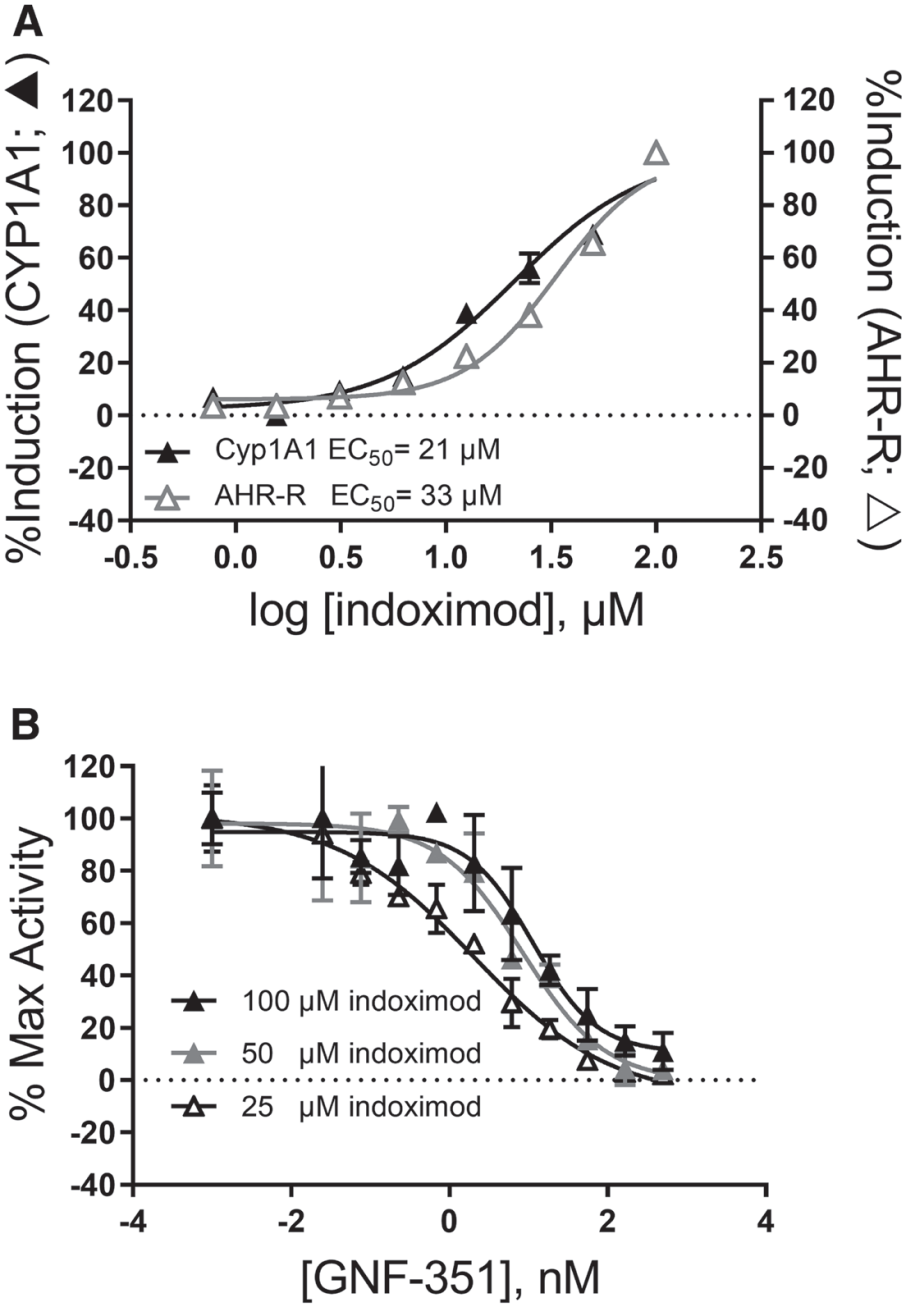
Indoximod can modulate transcription of AhR-controlled genes. (**A**) Representative plot of indoximod-induced expression and activity of endogenous CYP1A1 measured by EROD assay (black triangles) and that of luciferase controlled by an AhR-inducible promoter in HepG2 cells (open triangles). Data are representative from summary Supplementary Table 1 and Supplementary Table 2. (**B**) Indoximod-mediated transcriptional activation of AhR-controlled reporter gene is blocked by AhR inhibition in a competition assay between indoximod and GNF351. Representative plot of indoximod-induced expression and activity of luciferase driven by an AhR-inducible promoter in HepG2 cells. Data was normalized to the maximum induction at each indoximod dose level.

Toward that end, we examined the extent to which indoximod could directly influence AhR transcriptional activity in a luciferase reporter gene system and via the upregulation of CYP1A1 activity, a known AhR target gene. Data in Figure 4A shows that indoximod can modulate the transcription of reporter and endogenous genes containing AhR response elements in the promoter, inducing a 17-26-fold increase in transcription with an average EC_50_ of 33 ± 6 μM (Supplementary Table 1). Similarly, indoximod stimulates the expression and activity of CYP1A1 by 2–8 fold, with and EC_50_ of 23 ± 6 μM (Supplementary Table 2). Consistent with a mechanism in which indoximod acts via activation of the AhR pathway, the indoximod-driven AhR-reporter activity could be reverted by the AhR inhibitor GNF-351 in a dose dependent manner (Figure 4B). In conclusion, this data supports a model in which indoximod can behave as an AhR ligand and modulate the transcriptional activity of AhR on target genes.

### Indoximod changes the expression of AhR-controlled genes in activated human naïve CD4^+^ T cells

In addition to the role of Kyn/AhR axis in controlling expression of IDO in DCs, Kyn can drive the AhR-dependent differentiation of naïve CD4^+^ T cells into FoxP3^+^ regulatory T cells (Tregs) by controlling expression of the *FOXP3* gene [38, 41]. Additionally, other AhR ligands such as FICZ can skew CD4^+^ T cell differentiation towards an IL-17-producing helper T cell (TH17) phenotype [40]. Given our data suggesting that indoximod can modulate the transcription of genes containing AhR response elements in their promoter, and counteract the Kyn-mediated transcription of *IDO1* in moDCs, we hypothesized that indoximod’s mechanism of action might include opposing Kyn/AhR-driven effects on the CD4^+^ T cell differentiation program. Therefore, we investigated whether indoximod was able to modulate expression of *FOXP3* and *RORC*, the two main genes involved in the control of CD4^+^ T cell differentiation towards a Treg or TH17 phenotype, respectively. We studied this hypothesis in the context of a human CD4^+^ T-cell differentiation assay, where CD4^+^ T cells were stimulated with anti-CD3/anti-CD28 in the presence of IL-2 to promote their differentiation into Treg and/or TH17 cells [59].

**Figure 5:**
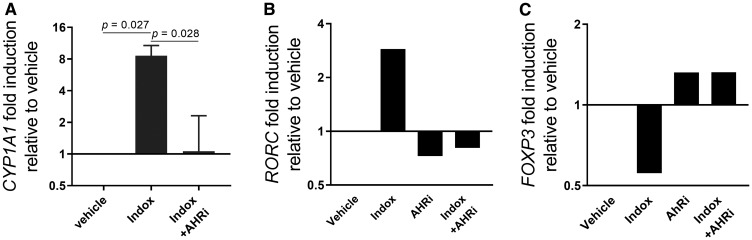
Indoximod regulates transcription of AhR-controlled genes in CD4^+^ T cells. Human CD4^+^ T cells were stimulated with anti-CD3/CD28 to promote their differentiation to Treg and Th17 cells. (**A**) Cells were cultured in the absence or presence of indoximod (100 μM) and/or the AhR inhibitor GNF351 (500 nM) for 5 h and analyzed for expression of *CYP1A1* mRNA by qRT-PCR. (**B**–**C**) human CD4^+^ T cells were stimulated for 4 days with anti-CD3/CD28 in the presence of Kyn (100 μM), indoximod (100 μM), and/or the AhR inhibitor CH223191 (10 μM) as indicated, and analyzed by qRT-PCR for expression of *RORC* (B) and *FOXP3* (C) mRNA transcripts.

The addition of indoximod to primary human CD4^+^ T cells in a differentiation assay stimulated AhR activity, as evidenced by the induction of *CYP1A1* mRNA (Figure 5A). These effects were reverted by an AhR inhibitor (CH113191 or GNF351) suggesting that indoximod was inducing the expression of the *CYP1A1* gene by directly or indirectly interacting with the AhR transcription factor. Additionally, indoximod induced upregulation of *RORC* expression while concurrently downregulating transcription of *FOXP3*, a gene that has also been shown to be regulated by AhR-ligand complexes [40] (Figure 5B and 5C). Both transcriptional effects of indoximod on *RORC* and *FOXP3* were reverted by an AhR inhibitor, confirming that indoximod appears to be modulating the transcription of these genes in an AhR-dependent fashion. Therefore, indoximod is capable to alter the ratio of *RORC*:*FOXP3* mRNA expression in CD4^+^ T cell cultures, thereby favoring differentiation into activated helper T cells rather than regulatory T cells.

### Indoximod blocks differentiation of naïve CD4^+^ T cells to Tregs while promoting differentiation to TH17 helper T cells

**Figure 6:**
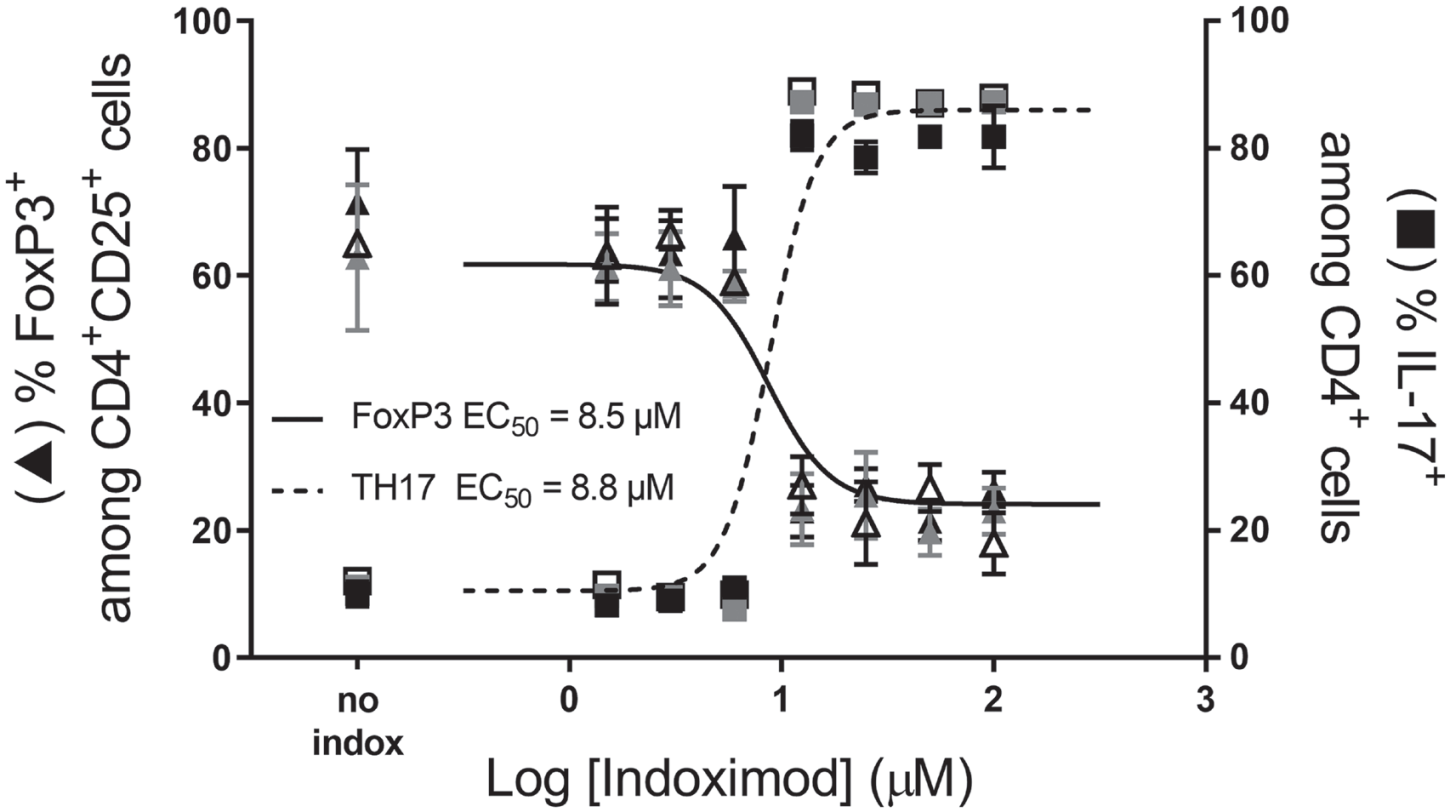
Indoximod favors CD4^+^ T cell differentiation to a TH17 helper phenotype while inhibiting differentiation to a Treg phenotype. A representative experiment demonstrating the influence of different concentrations of indoximod on CD4^+^ T cell differentiation in the absence (open symbols) or presence of Kyn (50 μM grey symbols, 100 μM, black symbols) to enhance their differentiation into Treg cells. Human CD4^+^ T cells were stimulated for 5 days with anti-CD3/CD28. The fraction of CD4^+^ CD25^+^ FoxP3^+^ (i.e., Treg cells) is shown on the left *y* axis (triangles) and the fraction of Th17^+^ T cells is represented on the right *y* axis (squares). Representative data selected from Table 1 Exp 45.

To complement the observation that indoximod can drive alterations in the ratio or RORC: FOXP3 mRNA in differentiating CD4^+^ T cells, we examined indoximod’s influence on the phenotype and function of differentiating CD4^+^ T cells at the protein level. Consistent with the indoximod-induced modulation of genes associated with T helper and Treg phenotypes, indoximod shifted the cellular phenotype away from FoxP3^+^ regulatory T cells and toward Th17-producing CD4^+^ helper T cells, thus driving differentiation in the opposite direction as that mediated by Kyn. Interestingly, the shift of differentiation from the Treg to Th17 phenotype takes place at the same EC_50_ (~4–9 μM), suggesting the existence of a common mechanism that affects both outcomes. A summary of several differentiation experiments performed with CD4^+^ T cells from different donors, and in the presence of different concentration of Kyn, indoximod and AhR inhibitors is presented in Table 1 and a representative set of experiments is shown in Figure 6.

**Table 1:** Summary of indoximod effect on Treg and TH17 differentiation

Exp^#^	Donor	Kynurenine (μM)	Indoximod range (μM)	Treg EC_50_ (μM)	TH17 EC_50_ (μM)
34	18	0	0.1–100	5.4	—
35	18	0	0.1–100	8.4	7.6
37	18	0	0.1–100	18.4	1.4
40	18	0	0.8–100	6.0	5.8
44	11	0	0.4–100	9.1	5.8
45	15	0	0.4–100	8.6	8.5
61	15	0	0.4–100	7.4	—
61	18	0	0.4–100	6.2	—
62	11	0	0.4–100	11.7	—
62	17	0	0.4–100	5.3	—
Combined		0		8.6 ± 4.0	5.8 ± 3.1
34	18	12.5	0.1–100	10.5	—
35	18	12.5	0.1–100	8.1	7.9
37	18	12.5	0.1–100	8.3	1.9
40	18	12.5	0.8–100	8.5	3.8
Combined		12.5		8.9 ± 1.1	4.3 ± 3.3
34	18	25	0.1–100	4.8	—
35	18	25	0.1–100	8.4	10.6
37	18	25	0.1–100	17.5	11.3
40	18	25	0.8–100	6.2	5.0
Combined		25		9.2 ± 5.7	9.0 ± 3.5
34	18	50	0.1–100	7.2	—
35	18	50	0.1–100	7.9	10.3
37	18	50	0.8–100	10.3	2.0
40	18	50	0.8–100	4.7	2.8
44	11	50	0.4–100	8.3	8.1
45	15	50	0.4–100	7.9	8.6
Combined		50		7.7 ± 1.8	6.3 ± 3.7
34	18	100	0.1–100	9.4	—
35	18	100	0.1–100	8.6	9.1
37	18	100	0.1–100	6.7	2.5
40	18	100	0.8–100	3.5	5.6
44	11	100	0.4–100	10.6	6.6
45	15	100	0.4–100	9.4	8.3
61	15	100	0.4–100	2.8	—
61	18	100	0.4–100	0.8	—
62	11	100	0.4–100	3.1	—
62	17	100	0.4–100	5.3	—
Combined		100 μM		6.0 ± 3.4	6.4 ± 2.6

### Indoximod opposes the Kyn-driven regulatory T cell differentiation of naïve CD4^+^ T cells

**Figure 7:**
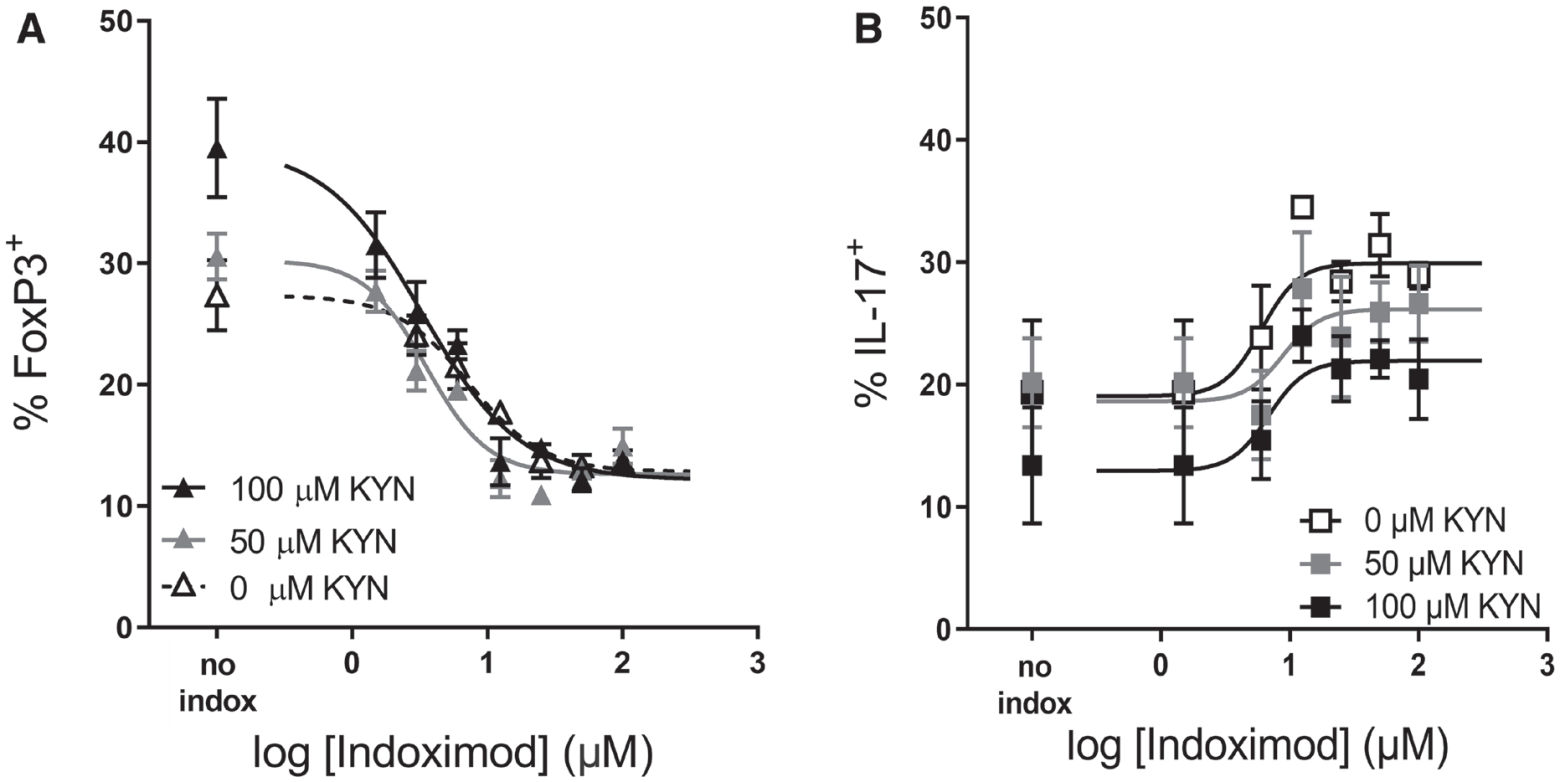
Indoximod opposes Kyn effects on Treg and TH17 differentiation. Human CD4^+^ T cells were stimulated for 5 days with anti-CD3/CD28 in the absence or presence of Kyn (50 or 100 μM) to enhance CD4^+^ T cell differentiation into Treg cells. Plot shows the influence of indoximod on the differentiation of CD4^+^ T cells into Treg (**A**) or TH17 helper T cells (**B**). Representative data selected from Exp 40 and 44 (Table 1).

To examine the influence of Kyn on Treg differentiation, Kyn was added at varying concentrations (0, 12.5, 25, 50 and 100 μM) to the indoximod titrations (Table 1). In general, the addition of exogenous Kyn enhanced Treg differentiation at concentrations of indoximod lower than 5 μM. Figure 7 shows an example in which the addition of exogenous Kyn in the absence of indoximod enhances the differentiation of Tregs (from 27% to 40% at 100 μM Kyn), while concurrently reducing the fraction of CD4^+^ T cells that differentiate into TH17 helper cells (from 20% to 13%). Indoximod is able to counteract the effect of Kyn, as evidenced by the reduced frequency of Tregs and increased fraction of IL-17-producing cells even at high levels of Kyn. Interestingly, indoximod-driven modulation of CD4^+^ T cell differentiation occurs in the absence of IDO^+^ or TDO^+^ expressing cells and in the absence of exogenous Kyn. This suggests that indoximod has a mechanism of action and pharmacodynamic effect that is independent of the presence of Kyn, yet which is also able to oppose the effects of Kyn on the differentiation of T cells. Consistent with this model, and to discard endogenous sources of IDO activity in these T cells, epacadostat was unable to reduce the fraction of FoxP3^+^ Tregs in these differentiation assays (Supplementary Figure 5).

### Indoximod suppression of Treg differentiation is reversed by AhR inhibition

**Figure 8:**
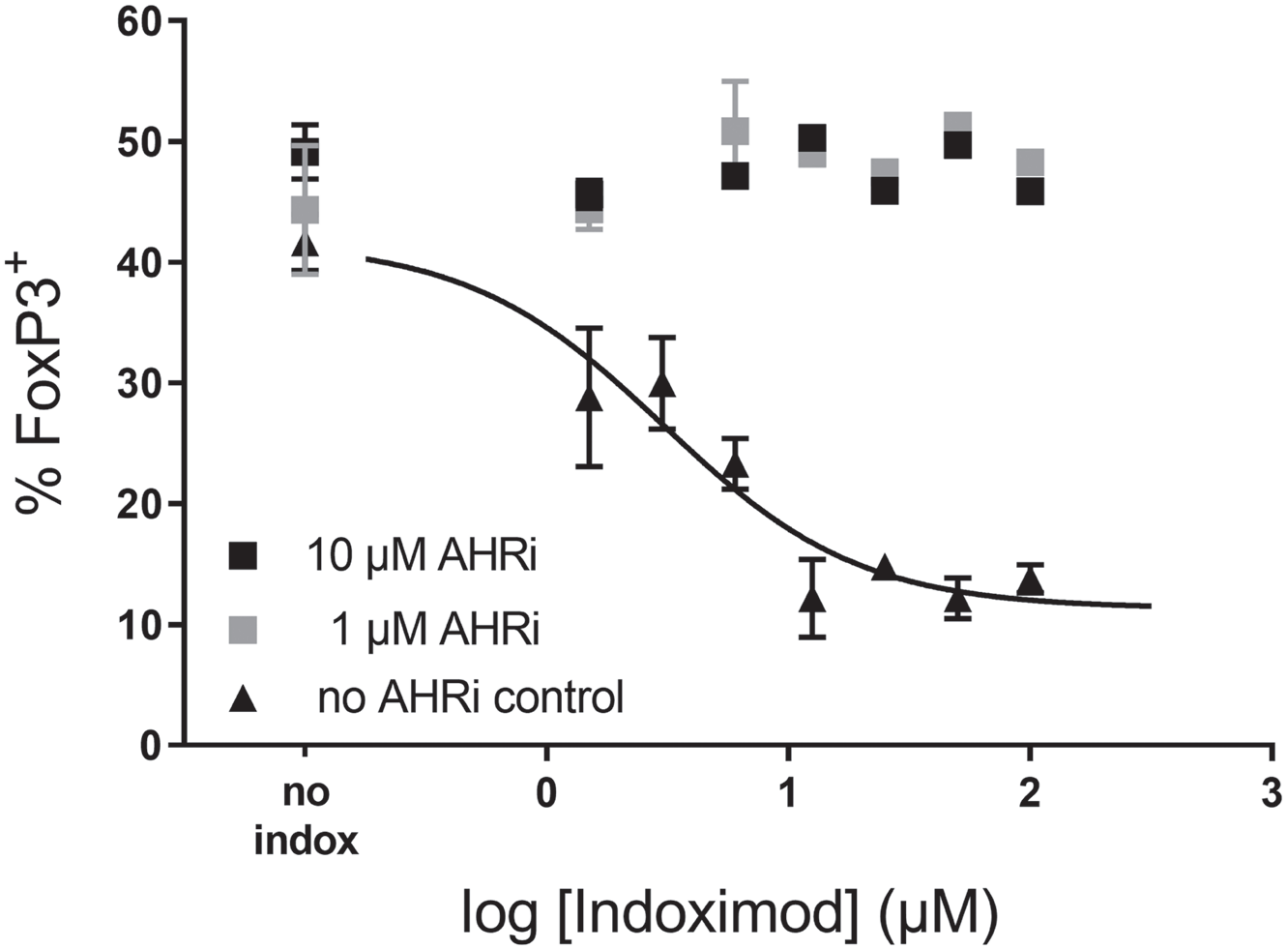
Indoximod effect on CD4+ T cell differentiation is inhibited by AhR inhibitors. Human CD4^+^ T cells were stimulated for 5 days with anti-CD3/CD28 in the presence of 100 μM Kyn to enhance their differentiation into Treg cells. Addition of the AhR inhibitor CH223191 (either 10 μM or 1 μM) reverses the effect of indoximod. Representative data selected from Exp 40 (Table 1).

To investigate the involvement of AhR activity in the indoximod-driven modulation of CD4^+^ T cell differentiation, we differentiated CD4^+^ T cells in the presence of 100 μM Kyn, a range of indoximod concentrations (0–100 μM) and 0, 1 or 10 μM of the AhR inhibitor CH223191. The data in Figure 8 demonstrate that the indoximod-driven reduction in the differentiation of FoxP3^+^ regulatory T cells can be blocked by inhibiting AhR. Similar results were observed using the AhR inhibitor GNF351 (at 500 nM) in the context of the differentiation assay. This suggests that indoximod changes the differentiation of CD4^+^ T cells by altering the transcriptional program driven by AhR, mainly by inhibiting transcription of *FOXP3* and by driving the transcription of *RORC*, thus opposing the transcriptional effects mediated by AhR/Kyn.

## DISCUSSION

Since the seminal work by Munn and Mellor that implicated tryptophan catabolism in the maintenance of an allogeneic fetus during pregnancy [4], and subsequent work that demonstrated that this pathway is also hijacked by tumors to promote immune evasion, IDO1 has been identified as a key player in mediating immune suppression to novel antigens [47, 48, 60]. In the studies that defined the importance of IDO1 in mediating immune suppression, the key compound used in defining the mechanism was the racemic compound 1-methyl-DL-tryptophan (1mT). This compound is a mixture of 2 enantiomers with different properties. The L isomer L1mT is a weak competitive inhibitor of IDO1 enzymatic activity and a mild substrate that can be converted to N-methyl-kynurenine, which could have potential immunosuppressive effects by signaling through AhR in a similar fashion as Kyn. On the other hand, the D isomer indoximod is not an inhibitor of the enzymatic activity of IDO1, nor is it a substrate. We have demonstrated in this work that indoximod has two mechanisms of action: 1) the creation of a Trp sufficiency signal; and 2) the modulation of AhR activity. Thus, caution should be taken when evaluating the biological effects of 1mT or indoximod and attributing those effects solely to the inhibition of IDO1 enzymatic activity. Indeed, much of the work on IDO and disease pathogenesis has relied on the use of indoximod, including important cancer studies. Therefore, a re-evaluation of the mechanistic implications derived from those studies should be carried out based on the evidence presented here.

In the extensive body of literature that precedes this work, it was shown that indoximod was able to relieve the inhibition of T cell proliferation mediated by the presence of IDO^+^ DCs, both *in vivo* (in TDLN), or *in vitro* in human or murine MLR cultures. These assays consistently suggested that the molecular target of indoximod was IDO expressed by the dendritic cell. This is still likely to be part of the pharmacological mechanism of indoximod, as shown here by the down regulation of IDO protein expression in pDCs and moDCs. However, additional effects of indoximod shown here demonstrate that indoximod can directly stimulate the proliferation of CD8^+^ T cells by reactivating the function of mTOR that was inhibited by conditions of low Trp. Most importantly, indoximod seems to interact with a pharmacological target directly in the T cell. This suggests a mechanism that is consistent with the creation of a Trp-sufficiency signal, which results in reactivation of mTOR, likely via the prior reactivation of MAP4K3/GLK. The nature of the amino acid sensor proteins that mediate the creation of the Trp-sufficiency signal are still not identified, but may involve or have analogs to the amino acid sensing complexes represented by the Rag GTPase, Gator, Castor or Sestrin proteins [61–69].

Indoximod, therefore, can restore mTOR activity under conditions of Trp-insufficiency created by the activities of either IDO or TDO, and thus mediate a direct stimulation of the proliferative capacity T cells. Stimulation of mTOR in T cells under conditions of Trp starvation required concentrations of indoximod in the same range as was required to stimulate T cell proliferation (~20–40 μM). These concentrations are much higher than was reported by Metz et al. in insulin-stimulated MCF7 cell assay, where the estimated EC_90_ was ~200 nM [37]. This apparent shift in effective potency may reflect assay-specific differences, or it may point to a difference in the amino acid sensing mechanisms available in tumor cell lines versus those present in T cells. Since this effect is observed at concentrations of indoximod of approximately 40 μM, the extent to which it contributes to the relief of immunosuppression created by IDO (and/or TDO) in the preclinical and clinical setting, is not clear. For example, concentrations of indoximod of > 40 μM are observed in mice orally dosed at > 250 mg/kg bid but are rarely seen in patients dosed with indoximod at the clinical dose of 1200 mg bid, where the average plasma concentration at steady state is ~10 μM [70, 71].

Despite evidence of the direct stimulation of T cell proliferation by indoximod, indoximod treatment of T cells co-cultured with IDO^+^ dendritic cells of murine or human origin also results in a reduction of Kyn in those cultures. This suggests that indoximod has a direct pharmacologic effect on the IDO-expressing cells, likely through downregulation of IDO protein.

Inflammation induced through vaccination drives IDO protein expression in pDCs in VDLN and TDLN, and indoximod administration during vaccination reduces IDO protein expression. This indoximod-driven reduction in IDO protein is consistent with the reduction of Kyn that is observed in *in vitro* MLR culture systems that utilize dendritic cells differentiated and matured *in vitro* to express active IDO protein. We recapitulated these observations in an *in vitro* model of human dendritic cells differentiation that favors expression of IDO protein. In this system, Kyn upregulates and indoximod downregulates expression of IDO protein (by FACS and western blot) when added during the differentiation and maturation phases. The indoximod-driven downregulation of IDO in moDCs has functional consequences, as Kyn production is also reduced and allogeneic T cell proliferation is enhanced by indoximod-treated DCs. Moreover, the indoximod-driven downregulation of IDO is blocked by GNF-351, suggesting that AhR is involved in mediating the pharmacologic effects of indoximod.

The exact molecular mechanism by which indoximod could downregulate expression of IDO in an AhR-dependent manner could have several explanations, particularly when considered in the context of other data available in the literature. The work by Li *et al*. [43] proposes that transcription of IDO is regulated by a positive feedback loop which requires the production of Kyn by IDO (since inhibition of IDO activity by 1mT downregulates IDO transcription) and the presence of AhR (since Kyn is not able to rescue IDO induction in AhR-deficient cells). Even though we used a different source, as well as a different differentiation/maturation protocol to obtain IDO-expressing DCs, our data are consistent with their model. We observe that Kyn is capable of inducing expression of IDO, and indoximod counteracts that effect. However, the indoximod-driven decrease in IDO protein expression does not appear to be driven solely by disruption of the IDO-kynurenine/AhR-IDO positive feedback loop, as the addition of the enzymatic inhibitor epacadostat during the differentiation did not result in a similar decrease in IDO protein expression. Further, we observe that AhR inhibitors prevent indoximod-mediated downregulation of IDO, while Li et al. shows that IDO cannot be induced by Kyn in DCs derived from spleens of AhR-deficient mice.

In addition to subtle differences in the differentiation protocols, the inherent complexity of IDO transcriptional regulation and the mechanistic diversity of AhR-ligand interactions, which is dependent on other transcription factors and has genomic as well as non-genomic mechanisms of action, could contribute to the mechanistic explanations for indoximod’s influence on IDO expression in dendritic cells. IDO transcriptional regulation is complex and subjected to both INFγ-dependent and INFγ-independent regulation [72]. The presence of IFNγ has a diversity of effects, which range from transient and biphasic induction of IDO [43] to downregulation of IDO when added to already matured IDO^+^ DCs [26]. Moreover, differences could be introduced by the concomitant use of other cytokines (i.e., IL-12 and sCD40L) in the differentiation and maturation of DCs, which could render the IDO promoter susceptible to a different mechanism of regulation. Moreover, INFγ also induces transcription of AhR in this model, which adds a layer of complexity to the regulation of IDO transcription. The data by Li *et al*. shows that AhR is not required for the initial induction of IDO by IFNγ but is required for the long-term maintenance of IDO expression. The mechanism by which Ahr/Kyn promotes transcriptional induction of the IDO1 promoter is not clear since there are no DRE/XRE consensus sequences in the proximal IDO1 promoter (though there is one DRE consensus sequence 5′-TNGCGTG-3′ 5.9 kb upstream of the human IDO1 gene). However, in BMDCs AhR ligands such as TCDD, FICZ, and Kyn are capable of inducing IDO1 mRNA in AhR-WT but not in AhR-null cells. This provides direct evidence that AhR can mediate transcriptional control of the IDO1 promoter despite the lack of DRE/XRE consensus sequences. This is not surprising, since AhR has been shown to associate to other transcription factors and bind to non-DRE consensus sequences [73, 74]. For example, AhR dimerizes with RelA and RelB leading to its recruitment to NF-κB responding sites [75, 76], which are present in the IDO1 promoter [72]. Under the AhR-dependent IDO1 transcriptional control model, it could be hypothesized that indoximod competes with Kyn to form a non-functional AhR complex, which dominantly blocks its transcriptional promoting function and/or its capacity to interact with other transcription factors. Under this model, a high affinity AhR inhibitor such as GNF351 would sequester AhR, prevent indoximod from dominantly exerting the transcription suppression of the promoter, and leave the transcription of IDO1 to the control of transcription factors that signal via the IFNγ-dependent (NF-κB or AP1) or IFNγ-dependent (IRF-1 or Stat1) pathways.

Alternatively, non-genomic functions of AhR could explain the indoximod-driven modulation of IDO1 protein in dendritic cells, perhaps through SOCS3-mediated degradation of IDO1. In its inactive state in the cytosol, AhR is complexed to HSP90, p23, AIP, and the c-SRC kinase [40]. Upon ligand binding, the c-SRC kinase is released from this complex and is capable of phosphorylating multiple c-SRC target proteins containing ITIM motifs [77, 78], such as those present in IDO1. Phosphorylated IDO1 is an enzymatically active protein, but becomes a substrate of SOCS3, a protein that mediates recruitment of E3 ubiquitin ligases and mediates ubiquitination of IDO1, thus promoting its proteasomal degradation [79, 80]. In addition, AhR has been shown to promote the proteasomal degradation of proteins such as estrogen and androgen receptors by recruitment of Cul4B ubiquitin ligase, in a ligand-dependent fashion [81–83]. Moreover, AhR can mediate the transcriptional activity of IL-6 and SOCS3, which are the key controllers in regulation of IDO1 enzyme. Thus, as suggested by Pallotta et al. [84], it is possible that AhR has a ligand-dependent genomic function as a canonical transcription factor capable of inducing IDO1, as well as IDO1-modulating factors (IL6 and SOCS3); at the same time, Ahr has a non-genomic role promoting the release c-Src-mediated phosphorylation of IDO1 while possibly promoting its ubiquitination by recruiting a hypothetical E3 ligase. Under this model, indoximod would bind to cytosolic AhR, promote c-Src mediated phosphorylation of IDO1 and AhR-mediated ubiquitination of IDO1, thus favoring its proteasomal degradation. The presence of a potent AhR inhibitor would prevent Ahr-mediated ubiquitination of IDO1, thus explaining the elevated levels of IDO protein observed under these conditions, despite the presence of indoximod. An additional possibility could be related to the creation of a Trp-sufficiency signal that results in activation of mTOR, which could further result of STAT signaling and activation of SOCS3 [85]. Under this model, indoximod would promote both mTOR activation in DCs that would result in increased SOCS3 activity, and Ahr-dependent c-Src phosphorylation of IDO1, both of which would contribute to enhanced IDO1 protein degradation.

The previous models assume that indoximod could directly interact with AhR, interfere with its interaction with Kyn, and modulate its genomic and/or non-genomic functions. We have not been able to demonstrate a direct molecular interaction between AhR and indoximod in biochemical studies based on surface plasmon resonance, competitive ligand equilibrium dialysis, or differential scanning calorimetry. These studies have been limited by technical difficulties in obtaining full length and pure preparations of AhR protein as well as the expected low binding affinity of indoximod to this target. Nonetheless, evidence of indoximod inducing the transcriptional activity of AhR was obtained in hepatic cell lines by measuring induction of the endogenous *CYP1A1* gene and by measuring expression of a luciferase reporter gene under the control of a minimal DRE promoter element. We observed that indoximod was able to induce those promoters and that its effect was competitively inhibited by the potent AhR inhibitor GNF-351. The activity of indoximod as a ligand of AhR had a potency of approximately 20–30 μM, which is similar to the measured EC_50_ binding affinity of Kyn for AhR by competitive binding of [^3^H]-TCDD in liver extracts [86]. These results agree with those presented by Moyer [51] and support the hypothesis that indoximod is a ligand of AhR. Additional support for a direct AhR-dependent transcriptional effect of indoximod was observed in our studies of the differentiation of primary human CD4^+^ T cells. In this assay, we observed that indoximod could induce the transcription of the AhR-regulated gene *CYP1A1*, as well as that of *RORC*, while simultaneously suppressing the transcription of *FOXP3*. These transcriptional effects of indoximod were reversed by the AhR inhibitors CH113191 or GNF351, which confirmed the involvement of AhR in mediating the pharmacologic effects of indoximod. Moreover, these transcriptional effects of indoximod were translated to phenotypic changes, reflected as an increase in IL-17 expressing helper T cells and a reduction in FoxP3^+^ Treg cells. In this assay, we confirmed that Kyn increased the differentiation of FoxP3^+^ regulatory T cells while decreasing the fraction of TH17-producting helper T cells. These Kyn-driven effects were dominantly reverted by indoximod, with an EC_50_ of ~8 μM regardless of the concentration of Kyn present during differentiation.

The influence of AhR and various AhR ligands on the differentiation of primary CD4^+^ T cells to regulatory or TH17 helper cells has been extensively studied [38, 41, 45, 87]. In these assays, TCDD and Kyn favor differentiation to a Treg phenotype (in the presence of TGFβ) while FICZ favors differentiation to a TH17 phenotype (in the presence of TGFβ and IL-6) [45]. These effects were demonstrated to be AhR-dependent since they do not take place in T cells from AhR-deficient mice or in the presence of an AhR inhibitor [45, 87]. We demonstrate that similarly to FICZ, indoximod has a TH17 polarizing effect, even under conditions that do not normally favor TH17 polarization (i.e., in the presence of IL2 and absence of TGFβ and IL6). Since TGFβ is an inducer of AhR mRNA, it is possible that the ligand-dependent AhR-polarizing effects described in other studies are exacerbated in that system [38] compared to the absolute effects that we observe in the absence of TGFβ.

The indoximod driven modulation of CD4^+^ T cell differentiation in our *in vitro* assays are consistent with the observations by Vogel, et al. in which the 1mT treatment of mice caused a reduction of Treg in the spleens of mice that had been dosed with TCDD to induce Treg differentiation [46]. They interpreted this effect as an AhR/TCDD-mediated induction of IDO in DCs, which drove differentiation of T cells into FoxP3^+^ Tregs, since inhibition of IDO with 1mT reverted the effect. However, the observation could also be explained by a direct effect of indoximod (present in racemic 1mT) on the differentiation of activated T cells.

Our observations that Kyn enhances T cell differentiation towards FoxP3^+^ and that indoximod opposes that effect is consistent with the observations by Mezrich, et al., who used a murine T cell differentiation assay carried out in the presence of TGFβ in low Trp medium (F10) [38], supplemented with TCDD or Kyn. This provides support to the hypothesis that Treg generation requires both Trp depletion and Kyn formation [28] as well as the function of AhR. Of note, indoximod can oppose both effects, as seen by mTOR reactivation in CD4^+^ and CD8^+^ T cells exposed to low Trp medium.

The addition of exogenous Kyn to the T cell differentiation cultures enhanced differentiation of Treg (i.e., a higher percentage of CD4^+^ cells were FoxP3^+^). Interestingly, indoximod potency on re-directing the Treg differentiation pathway was the same regardless of the concentration of Kyn. It was also independent from the presence of IDO or TDO expressing cells, suggesting that this is a Kyn-independent effect of indoximod; and while it opposes the differentiation promoted by Kyn, it supports a model in which indoximod is a fundamentally different class of inhibitor of the IDO/TDO pathways.

Indoximod appears to exert a reciprocal regulation of the *FOXP3* and *RORC* genes, and both effects are abolished by the presence of an AhR inhibitor. This raises the question of whether indoximod is acting like a transcriptional AhR agonist for *RORC* and an AhR antagonist for *FOXP3*, or whether the effect is only on one gene and propagated by signaling or co-dependent transcriptional control pathways to the other gene. The fact that both effects take place at the same potency of indoximod suggests that these effects are associated to a common molecular event but does not distinguish among the above-mentioned possibilities. The co-dependent regulation of the *FOXP3* and *RORC* genes is very complex and involves signaling by multiple cytokines and transcription factors as well as a direct interaction between FoxP3 and RORγt proteins [88, 89]. The *FOXP3* promoter contains AhR binding motifs [41] and ROR response elements, whereas the murine RORγt promoter contains HIF1 and HIF2 binding motifs, suggesting AhR involvement since HIF1β (ANRT) is the nuclear binding partner of AhR. Also, FoxP3 is known to antagonize RORγt function by direct interaction, thereby inhibiting TH17 cell differentiation [89]. Furthermore, the murine IL-17 promoter contains FoxP3 binding sequence and RORγt binding sequences suggesting a dual control of the IL-17 promoter by FoxP3 and RorC among others. Based on these complex regulatory elements and on the co-dependent regulation of *FOXP3* and *RORC* genes, we theorize that indoximod may likely be affecting the transcription of *FOXP3*, inhibiting its transcription by interfering with AhR signaling. Subsequently, signals would then propagate and result in increased *RORC* mRNA and reflected as an increase in IL-17-producing T cells.

The present work also highlights important differences between indoximod and classic enzymatic IDO inhibitors such as epacadostat. First, addition of epacadostat to monocyte differentiation cultures resulted in dendritic cells with increased levels of IDO protein, despite the reduction in Kyn synthesis. Thus, simply disrupting the IDO/Kyn/AhR maintenance loop does not appear to be sufficient to downregulate IDO protein expression in dendritic cells. Given that IDO1 also has been implicated to have a non-enzymatic signaling function that contributes to its self-amplification and results in the maintenance of a stable regulatory phenotype in pDCs that contributes to long-term tolerance [90], it is important to consider that abolishing enzymatic activity of IDO1 might not be sufficient and that reduction of IDO protein expression levels might be needed to achieve therapeutic benefit. This suggests that despite reducing production of Kyn, epacadostat may act as an agonist that induces IDO1 gene expression, thus self-limiting its efficacy as an IDO1 inhibitor, and this might be another factor that could explain the lack of activity of epacadostat observed in ECHO-301 melanoma clinical trial [91]. Furthermore, indoximod has IDO and TDO-independent T cell specific effects which lead to an increase in the proliferation of effector T cells and in the reprogramming of CD4^+^ T cell differentiation to a helper phenotype. These indoximod-driven T cell effects are not shared by IDO1-selective inhibitors.

In summary, indoximod has dual mechanistic effects that are based on reactivation of mTOR and on the modulation of AhR function. These effects are independent on the Trp metabolizing activity of IDO and/or TDO but happen to oppose the effects of the enzymatic activity of IDO and TDO by multiple mechanisms that act on cell types commonly affected by the IDO and TDO pathways. First, indoximod creates a Trp-sufficiency signal which leads to reactivation of MAP4K3 which leads to activation of mTORC1 activity, thus opposing and bypassing the effects of Trp deprivation that lead to GCN2 activation and MAP4K3 and mTOR inactivation. This effect requires a relatively high concentration of indoximod (approximately 20 μM under normal Trp levels and 45 μM under low Trp conditions), is observed in both CD4^+^ and CD8^+^ T cells and leads to an increase in the proliferative capacity of activated effector and helper T cells. Second, indoximod has AhR-dependent modulatory effects which affect the differentiation program of primary CD4^+^ T cells, favoring a TH17 helper phenotype and inhibiting the acquisition of a regulatory phenotype. This effect takes place at clinically relevant concentrations of indoximod (~5–9 μM) and is independent of IDO/TDO activity or exogenous Kyn, though it happens to oppose the Kyn/AhR effects on T cell differentiation. Third, indoximod can downregulate IDO1 protein expression in dendritic cells, with potency of approximately 20 μM, via a mechanism that involves the function of AhR (transcriptional or non-transcriptional) thus exerting a true IDO pathway inhibitory function, by blocking both the enzymatic and non-enzymatic signaling functions of IDO1 that contribute to T cell anergy, to the reduction of effector T cell proliferation, and to the promotion of regulatory T cell activation and differentiation. The different potencies at which these different effects are observed argue against the existence of a unifying underlying mechanism and suggest that mTOR reactivation and AhR modulation may be independent mechanisms. Together, these observations suggest that indoximod is a molecule with fundamentally different mechanism of action from IDO1-selective enzymatic inhibitors, which may have the clinical advantages of dual IDO/TDO inhibitors and warrants further exploration in clinical applications.

## MATERIALS AND METHODS

### Reagents

Indoximod free base was obtained from Sigma (Purity > 96%). Indoximod HCl was manufactured by Patheon (purity > 99.5%). Epacadostat was purchased from SelleckChem. Kyn, L-Trp, formic acid (FA), LCMS grade Water and LCMS grade Acetonitrile (ACN) were purchased from Fisher Scientific (USA).

### Purification of human CD8^+^ T cells

Human peripheral blood mononuclear cells (PBMC) were purchased from Cellular Technology Limited (CTL, Cleveland, OH, USA). For experiments using enriched CD8^+^ T cells, total PBMC were thawed and CD8^+^ T cells enriched using EasySep™ Human CD8^+^ T Cell Enrichment Kit (Stemcell Technologies Inc, Catalog # 17953). Magnetic separation was done with The Big Easy magnet (StemCell Technologies; Catalog #18001) as per manufacturer’s instructions. Purified CD8α^+^ cells were stained CFSE (carboxyfluorescein diacetate succinimidyl ester from Tonbo Biosciences; SKU: 13-0850) at a concentration of 5 μM per manufacturer’s protocol or with CellTrace Violet (Thermofisher) following manufacturer’s protocol.

### Human CD8^+^ T cell proliferation assay

In this assay, the human TDO-expressing SW48 cell line (colon adenocarcinoma) was cultured in RPMI 10% FBS supplemented with 200 μM of L-tryptophan, at 4 × 10^4^ cells/well in 96 V-bottom well plates, and incubated for 2 days in the presence of different concentrations of indoximod (0–100 μM). After 2 days of culture, CFSE-labeled CD8^+^ T cells obtained from human blood donors by magnetic negative selection were stimulated to proliferate with anti-CD3/CD28 beads and co-cultured (at 8 × 10^4^ cells/well) in the presence of the treated SW48 cells for an additional 72 hours. T-cell proliferation was measured by CFSE dilution by fluorescence-activated cell sorting (FACS) by gating on CD45^+^CD3^+^CD8^+^CD4^neg^ cells. In a variation of this experiment, CFSE-labeled CD8^+^ T cells were stimulated with CD3/CD28 beads and incubated in SW48-conditioned medium or in regular fresh RPMI medium in the presence of different concentrations of indoximod.

### *In vivo* IDO expression and T cell proliferation in a B16F10 tumor model involving gp100 vaccination and adoptive pmel-1 T cell transfer

Mice (C57Bl6) were injected subcutaneously with 10^5^ B16F10 murine melanoma cells. Doses of indoximod at 143, 287, 573, and 1147 μmol/kg/dose (equivalent to 31.3, 62.5, 125, 250 mg/kg/dose, respectively) were given bid by oral gavages (~12 h apart) from days 6 through 10 post tumor inoculation. The immunotherapy strategy included the adoptive transfer of 2 million dye-labeled tumor-antigen specific T cells isolated by negative magnetic selection (EasySep™ Mouse CD8^+^ T Cell Isolation Kit, StemCell Technologies; Catalog #19853) of CD8^+^ splenic T cells from transgenic pmel-1 mice. Isolated cells were stained with Cell Trace Violet before being administered by tail vein injection. Adoptively transferred CD8^+^ pmel-1 T cells [92] were activated by vaccination into the footpad with 50 μL of an emulsion of gp100 peptide (KVPRNQDWL, 25 μg), CpG ODN (from TriLink Biotechnologie Inc, 5′ TCC ATG ACG TTC CTG ACG TT 3′, 50 μg), and incomplete Freund’s adjuvant (IFA) on Day 7. In a variation of this study, the vaccination dose was modified by diluting the vaccine with PBS. On day 11, the vaccine-draining (popliteal) and tumor-draining (inguinal) LN were harvested and dissociated to create a single-cell suspension of each lymph node. Cells from individual lymph nodes were plated into 2 wells of a 96-well plate. Cells were blocked with anti-CD16/anti-CD32 and subsequently stained with anti-IDO (or isotype control), anti-B220, anti-CD45.1 and anti-CD11c. IDO1 protein levels in pDCs and the extent of T-cell proliferation was assessed by FACS analysis. Briefly, gating strategy was as follows: FSC vs SSC (live/lymphocytes) → FSC-H vs FSC-W (singlets) → CD11c vs CD317 (gating on pDCs, a.k.a. CD11c^+^CD317^+^) → determining the average magnitude of fluorescence intensity (gMFI) for IDO in the CD11c^+^CD317^+^ population or the percentage of the CD11c^+^CD317^+^ population that was positive for IDO expression (gated on isotype control antibody). For assessment of T cell proliferation, FCS files were analyzed using FlowJo 10.3 (FlowJo, LLC., USA) to gate specific populations and perform analysis of population proliferation. Gating strategy was as follows: FSC vs SSC (lymphocytes)→ FSC-H vs FSC-W (singlets)→ CD90.1 vs CD90.2 (gating on CD90.1^+^CD90.2^neg^ T cells)→ CD90.1 vs CD8 (gating on CD8^+^CD90.1^+^)→ proliferation analysis tool applied to CD8^+^CD90.1^+^ gate to calculate percent divided and division index, two parameters of cellular proliferation.

### *In vitro* differentiation of human monocyte-derived dendritic cells (moDCs)

The following *in vitro* culture system was used to differentiate human monocytes into moDC, and subsequently stimulated them to induce expression of functional IDO protein (Supplementary Figure 2) [26]. Briefly, purification of human IDO^+^ moDCs involves obtaining monocytes by the use of leukocytapheresis followed by counterflow elutriation. Monocytes were plated in RPMI 10% FCS media at 10^6^ cells/well in 24-well plates in the presences of IL-4 (500 U/mL) and GM-CSF (1000 U/mL), followed by culture for an additional 6 days. On Day 6, a maturation/stimulation cocktail was added to all wells, resulting in final concentrations of 1100 U/mL TNFα, 1870 U/mL IL-1β, 1000 U/mL IL-6, 1 μg/mL PGE2, 0.5 μg/mL anti-CD40 (clone 5C3) and 100 U/mL INFγ, followed by incubation for additional 48 h. On Day 8, the non-adherent fraction of cells was harvested and either used for assessment of IDO protein expression by western blot or by FACS or used in MLR assays with allogeneic T cells. The non-adherent fraction consists mostly of IDO^+^ moDCs (CD11c^+^, CD123^+^, CD83^+^, and express high levels of HLA-DR, CD80 and CD86). After culture, harvested moDCs were Fc blocked and stained for FACS analysis using the following antibodies: anti-IDO (ICS, clone eyeido, FITC), anti-CD86 (clone IT2.2, PE), anti-CD123 (clone 6H6, PerCP-Cy5), anti-CD40 (clone 5C3, PE-Cy7), anti-PDL1 (clone M1H1, APC), anti-CD11c (clone 3.9 APC-Cy7), anti-CD11b (clone M1/70, v450). For assessment of IDO expression by flow cytometry, FCS files were analyzed using FlowJo 10.3. Gating strategy was as follows: FSC vs SSC (live/lymphocytes) → FSC-H vs FSC-W (singlets) → CD11c vs CD123 (gating on moDCs, i.e., CD11c^+^CD123^+^) → determining the average magnitude of fluorescence intensity (gMFI) for IDO and isotype control in the CD11c^+^CD123^+^ population.

### T cell proliferation in MLR assays

MLR cultures were established by co-culturing 3.2 × 10^4^ IDO^+^ DCs with 8 × 10^4^ CD8^+^ allogeneic T cells (pre-stained with 5 μM CellTrace) and co-cultured in V-bottom 96-well plates for 5 days in RPMI 10% FBS (Supplementary Figure 2). After the MLR culture T cells were harvested, Fc blocked with anti-CD16/anti-CD32 and stained with anti-CD3, anti-CD45, anti-CD4 and anti-CD8α using the following antibodies: a) for CFSE dye: anti-CD3 (clone 145-2C11, PerCP-Cy5), anti-CD45 (clone 30-F11, APC), anti-CD4 (clone RM4-5, APC-Cy7), anti-CD8α (clone 53-6.7, v450); b) for Cell Trace Violet dye: anti-CD3 (clone 145-2C11, PerCP-Cy5), anti-CD4 (clone RM4-5, APC-Cy7), anti-CD8α (clone 53-6.7, v450). For assessment of T cell proliferation in MLR cultures, FCS files were analyzed using FlowJo 10.3. The gating strategy was as follows: FSC vs SSC (lymphocytes)→ FSC-H vs FSC-W (singlets)→ CD45 vs CD3 (gating on CD45^+^CD3^+^ T cells)→ CD4 vs CD8 (gating on CD8^+^CD4^neg^)→ proliferation analysis tool applied to CD8^+^CD4^neg^ gate to calculate percent divided and division index.

### Measurement of Kyn and Trp in culture supernatants

Kyn and Trp were analyzed using LC-MS/MS method. Sample were prepared by mixing 40 μL (sample, or calibration standards) with 4 vol acetonitrile, centrifuged 1640 × g for 5 min, and supernatant mixed with 2 vol of 0.1% formic acid in water. Calibration standards were prepared in the ranges of 30 nM to 100 μM in the same matrix as the sample. Analysis was carried out using UPLC BEH C18 column with a Waters Acquity UPLC system coupled to a TQD mass spectrometer equipped with a turbo-electrospray interface with positive ionization mode. The aqueous mobile phase was 0.1% formic acid in water and the organic mobile phase was 0.1% formic acid in acetonitrile (flow rate 0.6 mL/min). Quantitation of Trp and Kyn were carried out using multiple reaction monitoring (MRM), with following transition for Kyn 209.1→ 94.1 and for Trp 205.1→ 146.1.

### Measurement of AhR activity in AhR-luciferase reporter cell lines

Two lentiviral vectors were cloned by standard techniques: a) LKO-puro-luc2/XRE expressing firefly luciferase under the control of an AhR-dependent promoter containing XRE elements (pGL4.43[luc2p/XRE], Promega); and b) LKO-neo-Renilla-luc expressing *Renilla* luciferase under the control of the constitutive TK promoter. HepG2 cells lines were co-transduced with these lentiviral vectors and selected for resistance to both puromycin and G418. The resulting cell line expressed a constitutive luciferase and an AhR-inducible luciferase, the activity of which could be measured in the same assay sample by the use of different luminogenic substrates. AhR reporter activity was measured with Dual-Glo^®^ Luciferase Assay System (Promega, Cat.# E2940) after incubation of the cells with the test compounds for 6–18 h. The normalized AhR promoter activity value was calculated as the ratio of firefly luciferase to *Renilla* luciferase activity in each assay. Normalized activity values (in triplicate) were plotted vs. the logarithm of the concentration of the test compound in each well to determine EC_50_ values. Since the maximum fold-induction of the AhR promoter varies greatly when using different AhR ligands, the maximum fold-induction observed at 100 μM indoximod was used as the maximum value of the plateau in the equation parameters to determine the EC_50_ value. The inducibility of the AhR-dependent luciferase in this HepG2 cell line was confirmed by treatment of the reporter cell lines with different concentrations of TCDD, which produced a ~100-fold induction at 10 nM TCDD (not shown).

### Measurement of AhR activity by induction of CYP1A1 activity

The activity of the endogenous AhR-inducible *CYP1A1* gene was measured in HepG2 cells after incubation of these cells with the test compound for a period of 6–18 h. Subsequently, CYP1A1 activity was measured by assessing the conversion of the CYP1A1 substrate 7-ethoxyresorufin (2 μM) into resorufin by fluorescence detection (EROD assay; Ex: 535 nm, Em:590 nm). For data analysis, fluorescence values (in triplicate) were normalized to the cell number per well as determined by the luminescence value of the CellTiterGlo assay and plotted vs. the Log of the concentration of compound in each well (i.e., indoximod or TCDD). The maximum fold-induction observed at 100 μM indoximod was used as the maximum value of the plateau in the equation parameters to determine the EC_50_ value.

### CD4^+^ T cell differentiation assay

Human peripheral blood mononuclear cells (PBMC) were purchased from Cellular Technology Limited (CTL, Cleveland, OH, USA). CD4^+^ T cells were enriched using EasySep™ Human CD4^+^ T Cell Enrichment Kit (Stemcell Technologies Inc, Catalog # 17952). Magnetic separation was done with The Big Easy magnet (StemCell Technologies; Catalog #18001) as per manufacturer’s instructions. Thermo Scientific IMMULON II Flat Plate 96-well (Fisher 1424561LC) were coated with anti-CD3 antibody (clone: OKT3, Tonbo; 10 μg/mL in PBS) overnight at 4°C. Plates were washed once with RPMI/10% FCS media prior to plating T cells. Stimulation cultures of T cells included addition of anti-CD28 (clone: CD28.2, Tonbo; 1 μg/mL) and IL-2 (Tonbo; 100 U/mL) in complete RPMI media. Enriched CD4^+^ cells were spun down, plated at 8 × 10^4^ cells/well into anti-CD3-coated plates (with anti-CD28 and IL-2), and allowed to differentiate for 4–5 days in the presence of vehicle, indoximod (1.5–100 μM), Kyn (50–100 μM) and/or AhR inhibitor GNF351 (500 nM) or CH223191 (10 μM).

### Quantitative RT-PCR in CD4^+^ T cell differentiation assays

For qRT-PCR analysis, indoximod (100 μM); the AhR inhibitor GNF-351 (500 nM) or the AhR inhibitor CH223191 (10 μM); or both (indoximod and AhRi) were added to 15 wells of plated CD4^+^ T cells per condition, to allow for the collection of requisite quantity of RNA from each group for proper analysis. After 4 days of differentiation culture, T cells from each group were pooled, and RNA was purified using Qiagen RNeasy Kit. DNA contamination was removed from RNA preps using the DNAfree DNA Removal Kit (Invitrogen AM1906). cDNA was prepared using the iScript Reverse Transcription Supermix (BioRad). The indoximod-driven induction of expression for *CYP1A1*, *RORC*, *FOXP3*, and *GAPDH* was determined by SYBR Green qRT-PCR on a StepOnePlus™ Real-Time PCR System (Applied Biosystems™). Primer sequences were the following: *RORC*: 5′-TGA GAA GGA CAG GGA GCC A-3′; 5′-CCA CAG ATT TTG CAA GGG ATC-3′; *FOXP3*: 5′-TGC CTC CTC TTC TTC CTT GAA C-3′; 5′-TCC TGG AGG AGT GCC TGT AAG T-3′; *CYP1A1*: 5′-TCT TCC TTC GTC CCC TTC AC-3′; 5′-TGG TTG ATC TGC CAC TGG TT-3′; *GAPDH*: 5′-GAA GAC GGG CGG AGA GAA AC-3′; 5′CCA TGG TGT CTG AGC GAT GT-3′.

### Analysis of CD4^+^ T cell phenotype after differentiation assay

After differentiation culture, T cells from each well were stained for either FoxP3 or IL-17 expression by staining cells with fluorescently labeled antibodies for the surface and intracellular proteins expressed by cells. For FoxP3 expression analysis, T cells were Fc-blocked with anti-CD16/CD32, permeabilized and fixed, followed by staining with the following panel of antibodies: anti-FoxP3 (clone 236A/E7, PE), anti-CD3 (clone OKT3, PerCP-Cy5), anti-CD25 (clone BC96, APC) and anti-CD4 (clone RPA-T4, v450). For IL-17 and FoxP3 expression analysis, T cells were stimulated to produce cytokine by addition of PMA/ionomycin/brefeldin A (Tonbo, TNB-4975-UL100 Cell Stimulation cocktail), for 5 h, followed by washing, Fc-blocked with anti-CD16/ CD32, fixed, permeabilized and stained with the following panel of antibodies: anti-IL17 (clone eBio64DEC17, PerCP-Cy5), anti-FoxP3 (clone 236A/E7, PE), anti-CD3 (clone SK7, v450), anti-CD25 (clone BC96, APC) and anti-CD4 (clone RPA-T4, APC-Cy7). Stained samples were run on a FacsCantoII (BD Biosciences) running BD FACSDiva Software, Version 8.0.1. Files were exported for analysis as FCS 3.0 files. Gating strategy was as follows: FSC vs SSC (lymphocytes)→ FSC-H vs FSC-W (singlets) → CD3 histogram plot (gating on CD3^+^ T cells)→ CD4 vs CD25 (gating on CD25^+^CD4^+^)→ assessing the expression of FoxP3 or IL-17 in the CD4^+^CD25^+^ population. To determine the EC_50_ value of indoximod, the percentage of total FoxP3^+^ or IL-17^+^ T cells from each treatment condition (in triplicate) was plotted vs. the logarithm of the concentration of treatment compound in each well and non-linear regression using the dose-response with variable Hillslope function was carried out using GraphPad Prism.

## SUPPLEMENTARY MATERIALS



## Abbreviations

1mT: 1-methyl-DL-tryptophan
AhR: aryl hydrocarbon receptor
ARNT: aryl hydrocarbon receptor nuclear translocator
DC: dendritic cell
FACS: fluorescence activated cell sorting/scanning
gMFI: geometric mean fluorescence intensity
IDO: indoleamine 2,3-dioxygenase
Kyn: L-kynurenine
L1mT: 1-methyl-L-tryptophan
MLR: mixed lymphocyte response proliferation
moDC: monocyte derived dendritic cell
mTOR: mammalian target of rapamycin
TCDD: 2,3,7,8- tetrachlorodibenzo-p-dioxin
pDC: plasmacytoid dendritic cell
TDLN: tumor draining lymph node
TDO: tryptophan 2,3-dioxygenaseTrp: L-tryptophan
TH17: IL-17 producing helper T cell
Tregs: regulatory T cells
VAX: vaccine
VDLN: vaccine draining lymph node
