# Supplementary material for: Indoximod opposes the immunosuppressive effects mediated by IDO and TDO via modulation of AhR function and activation of mTORC1
Source: Oncotarget. 2020 Jun 23;11(25):2438–61. doi: 10.18632/oncotarget.27646 (PMC7321702; doi:10.18632/oncotarget.27646)
Supplement: Supplementary file 1 [file oncotarget-11-2438-s001.pdf]

Indoximod opposes the immunosuppressive effects mediated by IDO and TDO via modulation of AhR function and activation of mTORC1

SUPPLEMENTARY MATERIALS

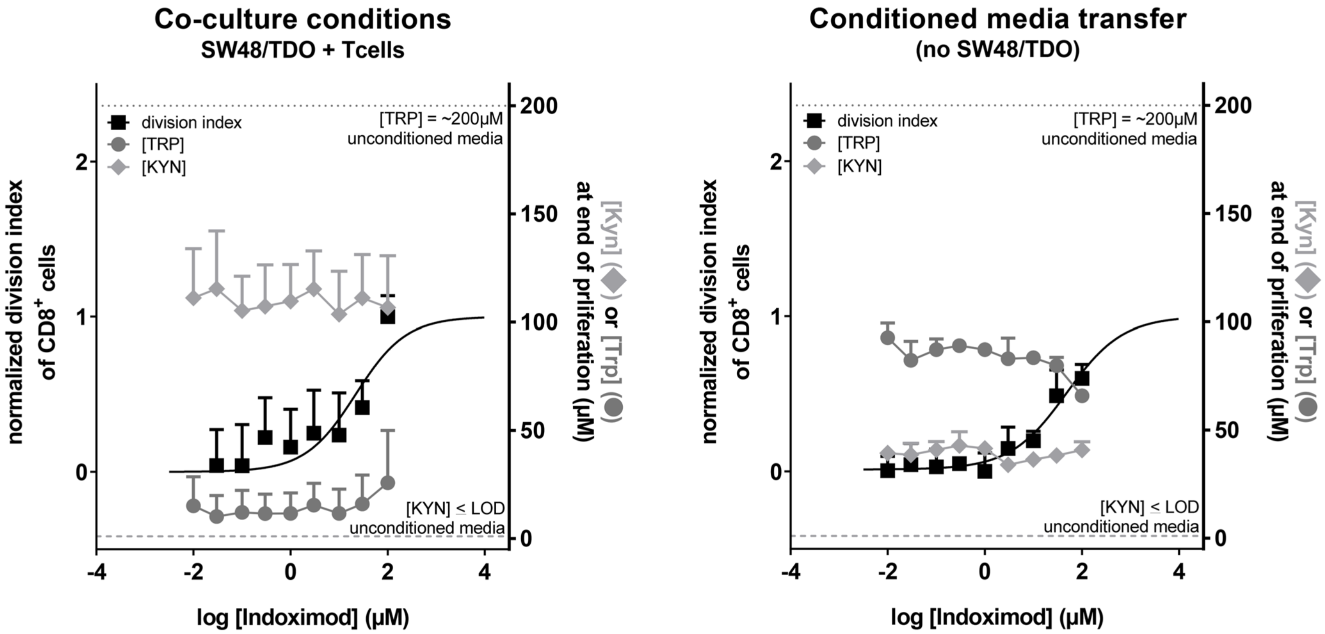

Supplementary Figure 1: Modulation of tryptophan and kynurenine by TDO is independent of indoximod concentration in CD8 T cell proliferation assays. Culture assay supernatants from the cultures described in Figure 1A were harvested at the end of the T cell proliferation period and the concentration of tryptophan and kynurenine was assessed by LCMS. The graph on the left displays proliferation data for T cells in direct co-culture with TDO-expressing SW48 cell (black squares, ■, left vertical axis). The graph on the right displays proliferation data for T cells cultured in media that had been conditioned for 48 hours by TDO-expressing SW48 cells. The tryptophan (dark gray circles, ●, right vertical axis) and kynurenine (light gray diamonds, ♦, right vertical axis) data have been included for the corresponding culture condition. The input concentrations of tryptophan and kynurenine (i.e., previous to SW48/TDO conditioning) have been indicated on the graphs as a dotted line at 200 µM for tryptophan and a dashed line to indicate the limit of detection (LOD) for kynurenine.

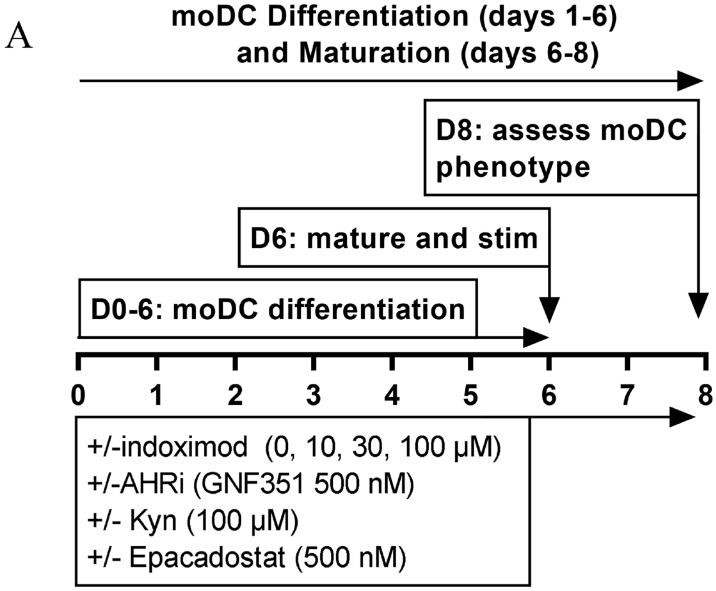

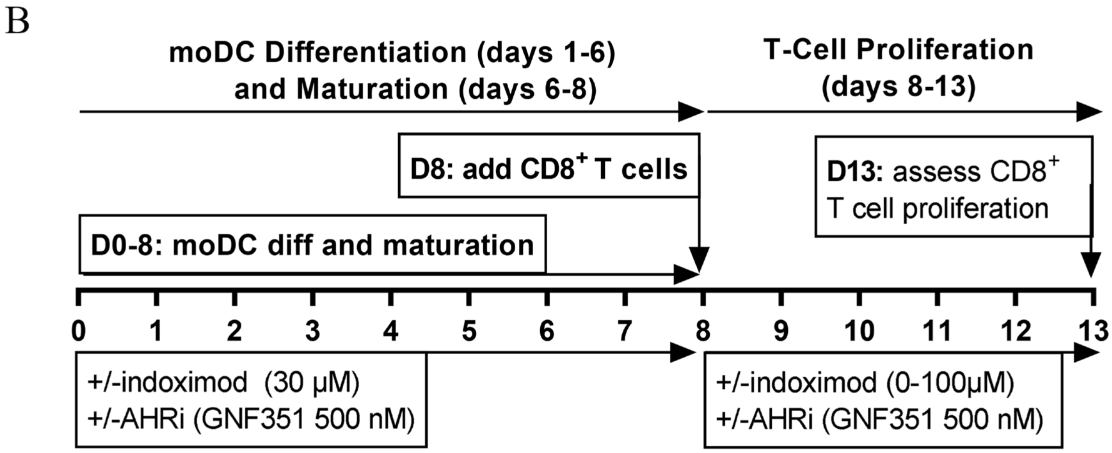

Supplementary Figure 2: Differentiation of protocol scheme of human monocyte-derived dendritic cells. Schematic protocol design for differentiation of human monocytes into IDO$^+$ moDCs. (**A**) Protocol to obtain IDO$^+$ moDCs for western blot or FACS analysis. Incubation with test agents indoximod, GNF352, Kyn and/or Epacadostat was initiated on Day 1 and extended until analysis on Day 8. Protocol details available in Methods section. (**B**) Human monocytes obtained by the same differentiation and maturation protocol as in A were harvested and co-cultured with dye-labeled allogeneic human CD8$^+$ T cells for 4 days, to assess the impact of treatment of moDCs during differentiation with indoximod or AhR inhibitors. In addition, indoximod and/or AhR inhibitor were added during the T cell proliferation phase. T cell proliferation was assessed on Day 13 by FACS analysis.

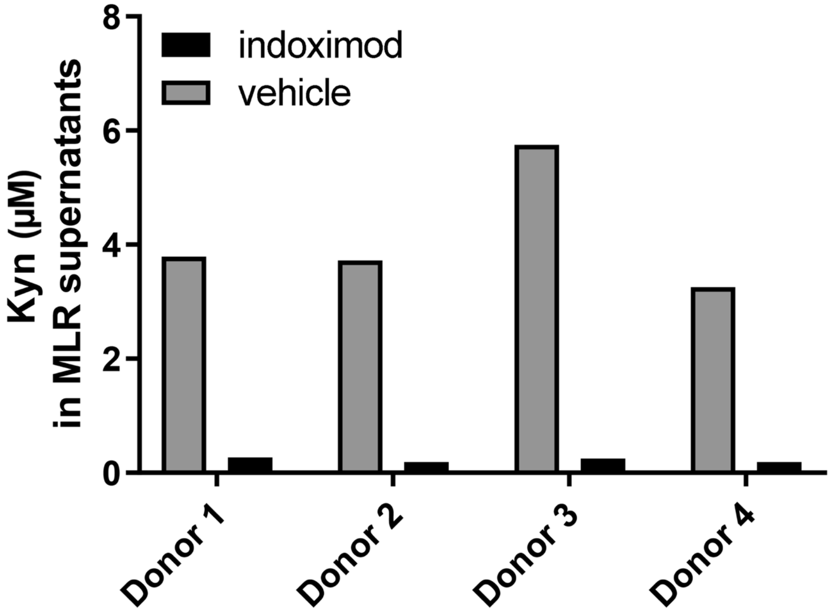

Supplementary Figure 3: Kyn production in MLR assays. Kyn synthesis in MLR assays. moDCs were obtained by leukocytapheresis from 4 different human donors, differentiated and matured according to the protocol of Supplementary Figure 1B, in the absence or presence of 30 µM indoximod. T cells from allogeneic donor pairs were co-cultured with these moDCs for 4 days and Kyn levels in the supernatant were measured by LC-MS/MS.

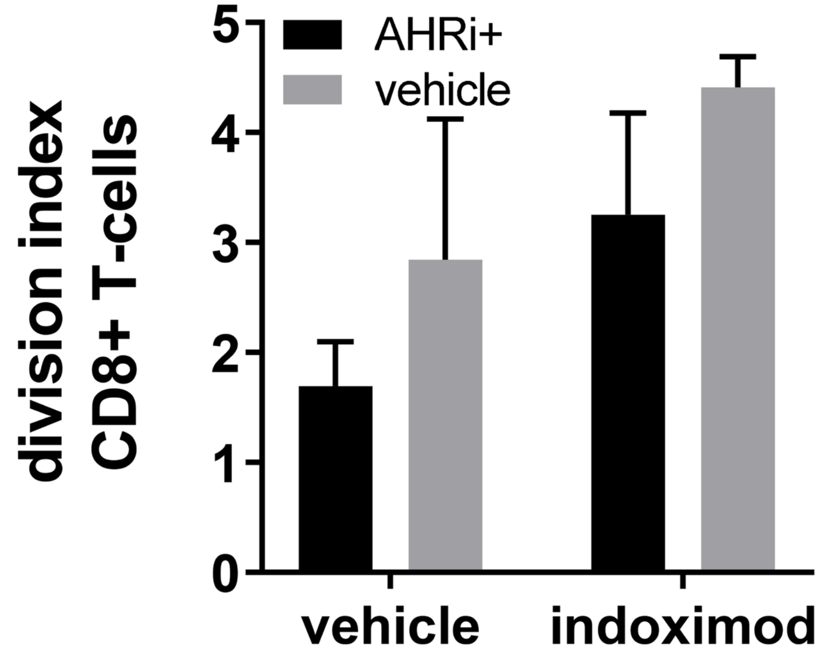

Supplementary Figure 4: AhR does not inhibit proliferation of CD8$^+$ T cells in the absence of IDO$^+$ moDCs. To investigate the inhibitor GNF-351 could inhibit proliferation of CD8$^+$ T cells, dye-labeled human primary CD8$^+$ T cells were stimulated with anti-CD3/CD28 and incubated in the absence and presence of 100 µM indoximod and in the absence or presence of 500 nM GNF351. At the end of the proliferation period, T cell proliferation was measured by FACS using the dye-dilution analysis tools in FloJo. There were no statistical differences between vehicle and AhR inhibitor, both in the absence or presence of indoximod indicating that GNF351 does not have a toxic or inhibitory effect on T cell proliferation.

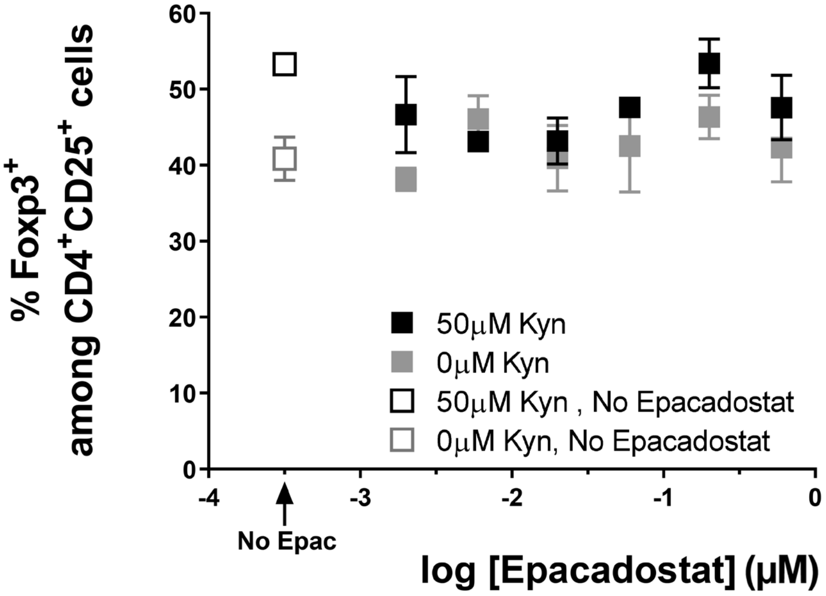

Supplementary Figure 5: Epacadostat does not affect differentiation of $CD4^+$ T cells. Epacadostat does not reduce the fraction of $FoxP3^+$ T cells obtained after a 4-day differentiation culture of primary human $CD4^+$ T cells stimulated with anti-CD3/CD28 and IL-2, in the absence or presence of 50 µM Kyn. For details see Materials and Methods.

Supplementary Table 1: Summary of AhR-luciferase induction by indoximod

Exp #	Indoximod Source	Solvent	Max fold induction	EC_{50} (µM)
LUC.1	Sigma lot 09009BE	DMSO/HCl	26	43.3
LUC.2	Sigma lot 09009BE	DMSO/HCl	22	24.2
LUC.3	Sigma lot MKBZ1441V	DMSO/HCl	17	33.6
LUC.4	Sigma lot MKBZ1441V	DMSO/HCl	21	35.2
LUC.5	Sigma lot MKBZ1441V	DMSO/HCl	21	32.3
LUC.6	Regis lot P235-42-2	DMSO/HCl	22	34.6
LUC.7	Regis lot P235-42-2	DMSO/HCl	23	33.7
LUC.8	Regis lot P235-42-2	DMSO/HCl	19	25.1
	Combined		17-26 fold	32.7 ± 6.0

Supplementary Table 1 shows independent experiments carried out with 3 different lots of indoximod, to assess induction of AhR-driven expression of firefly luciferase normalized to that of Renilla luciferase. Maximum fold induction is the ratio of normalized luciferase activity observed at 100 µM indoximod relative to the normalized luciferase activity in vehicle control. EC_{50} is the concentration of indoximod that is necessary to achieve half of the induction level observed at 100 µM indoximod. For details see Materials and Methods.

Supplementary Table 2: Summary of CYP1A1 induction by indoximod

Expt #	Source	Solvent	Max fold induction	EC_{50} (µM)
CYP.1	Sigma lot 09009BE	DMSO/HCl	3.2	13.1
CYP.2	Regis lot P235-42-2	DMSO/HCl	2.6	22.8
CYP.3	Sigma lot 09009BE	DMSO/HCl	5.2	20.2
CYP.4	Regis lot P235-42-2	DMSO/HCl	3.8	26.3
CYP.5	Sigma lot 09009BE	DMSO/HCl	8.3	25.6
CYP.6	Regis lot P235-42-2	DMSO/HCl	4.4	36.9
CYP.7	Sigma lot 09009BE	DMSO/HCl	3.1	19.5
CYP.8	Regis lot P235-42-2	DMSO/HCl	2.1	29.2
CYP.9	Sigma lot 09009BE	DMSO/HCl	2.8	17.8
CYP.10	Sigma lot MKBZ1441V	DMSO/HCl	2.5	21.2
CYP.11	Regis lot P235-42-2	DMSO/HCl	2.8	22.4
	Combined		2.1-8.3 fold	23.2 +/– 6.3

Supplementary Table 2 shows independent experiments carried out with 3 different lots of indoximod, to assess induction of CYP1A1 enzymatic activity, assessed by de-ethylation of 7-ethoxyresorufin into resorufin by a fluorescence assay (EROD assay). Maximum fold induction is the ratio of CYP1A1 activity observed at 100 µM indoximod relative to the activity in vehicle control. EC_{50} is the concentration of indoximod that is necessary to achieve half of the induction level observed at 100 µM indoximod. For details see Materials and Methods.
